# Dual Analysis of the Murine Cytomegalovirus and Host Cell Transcriptomes Reveal New Aspects of the Virus-Host Cell Interface

**DOI:** 10.1371/journal.ppat.1003611

**Published:** 2013-09-26

**Authors:** Vanda Juranic Lisnic, Marina Babic Cac, Berislav Lisnic, Tihana Trsan, Adam Mefferd, Chitrangada Das Mukhopadhyay, Charles H. Cook, Stipan Jonjic, Joanne Trgovcich

**Affiliations:** 1 Department of Histology and Embryology and the Center for Proteomics, University of Rijeka School of Medicine, Rijeka, Croatia; 2 Laboratory of Biology and Microbial Genetics, Faculty of Food Technology and Biotechnology, University of Zagreb, Zagreb, Croatia; 3 The Department of Surgery, The Ohio State University, Columbus, Ohio, United States of America; University of North Carolina at Chapel Hill, United States of America

## Abstract

Major gaps in our knowledge of pathogen genes and how these gene products interact with host gene products to cause disease represent a major obstacle to progress in vaccine and antiviral drug development for the herpesviruses. To begin to bridge these gaps, we conducted a dual analysis of Murine Cytomegalovirus (MCMV) and host cell transcriptomes during lytic infection. We analyzed the MCMV transcriptome during lytic infection using both classical cDNA cloning and sequencing of viral transcripts and next generation sequencing of transcripts (RNA-Seq). We also investigated the host transcriptome using RNA-Seq combined with differential gene expression analysis, biological pathway analysis, and gene ontology analysis.

We identify numerous novel spliced and unspliced transcripts of MCMV. Unexpectedly, the most abundantly transcribed viral genes are of unknown function. We found that the most abundant viral transcript, recently identified as a noncoding RNA regulating cellular microRNAs, also codes for a novel protein. To our knowledge, this is the first viral transcript that functions both as a noncoding RNA and an mRNA. We also report that lytic infection elicits a profound cellular response in fibroblasts. Highly upregulated and induced host genes included those involved in inflammation and immunity, but also many unexpected transcription factors and host genes related to development and differentiation. Many top downregulated and repressed genes are associated with functions whose roles in infection are obscure, including host long intergenic noncoding RNAs, antisense RNAs or small nucleolar RNAs. Correspondingly, many differentially expressed genes cluster in biological pathways that may shed new light on cytomegalovirus pathogenesis. Together, these findings provide new insights into the molecular warfare at the virus-host interface and suggest new areas of research to advance the understanding and treatment of cytomegalovirus-associated diseases.

## Author Summary

We have conducted a comprehensive analysis of the murine cytomegalovirus and host cell transcriptomes during lytic infection. We identify numerous novel spliced and unspliced transcripts of MCMV. Unexpectedly, the most abundantly transcribed viral genes are of unknown function. We found that the most abundant viral transcript, recently identified as a noncoding RNA regulating cellular microRNAs, also codes for a novel protein. To our knowledge, this is the first viral transcript that functions both as a noncoding RNA and an mRNA. Infection alters expression of many unexpected host genes, including many noncoding RNA genes. Correspondingly, many cluster in unexpected biological pathways that may shed new light on cytomegalovirus pathogenesis. Together, these findings provide new insights into the molecular warfare at the virus-host interface and suggest new areas of research to advance the understanding and treatment of cytomegalovirus-associated diseases.

## Introduction

The cytomegaloviruses, classified within the *Betherpesvirinae* subfamily, are a group of species-specific herpes viruses that establish life-long infection of their hosts. Human cytomegalovirus (HCMV) can cause devastating disease and death in congenitally-infected infants, and long-term neurological complications in survivors. In adults, HCMV can cause a spectrum of diseases in immune compromised patients involving multiple organs and tissues and is a primary cause of graft loss in transplant patients [1], [2]. In recent years, HCMV has been linked to lung injury in trauma patients [3] and is also postulated to act as a cofactor in atherosclerosis and some cancers [4], [5]. For these reasons, there is an urgent need for an effective vaccine and new antiviral intervention strategies that mitigate the toxicity and drug resistance shortcomings of current antiviral drugs [1], [6].

There exist a number of challenges to our understanding of CMV pathogenesis as well as progress in vaccine and antiviral drug development. Two outstanding challenges are the gaps in our knowledge of viral genes and how these gene products interact with host cellular gene products to cause disease. Despite the publication of the first sequence of the HCMV genome in 1990 [7], [8], and the first sequence of the murine cytomegalovirus (MCMV) genome in 1996 [9], there are still important questions regarding the nature and number of genes for these viruses.

MCMV is the most widely used model to study HCMV diseases and recapitulates many of clinical and pathological findings found in human diseases. Our understanding of MCMV viral genes and genomes has evolved with the technology used to study them. A major milestone in understanding MCMV came with decoding the first MCMV complete genome sequence by Rawlinson and colleagues [9]. The authors identified a 230 kb genome predicted to encode 170 genes.

Subsequent refinements in the annotation of the MCMV were introduced by classical molecular and biochemical studies that are reflected in the current NCBI reference sequence. The application of new technologies to study the MCMV genome emerged in the last decade and include proteomic [10], in silico [11], and gene array [12], [13] approaches that have led to major revisions in gene annotation. More recently Cheng and colleagues [14] proposed additional changes after sequencing isolates to measure genome stability after *in vitro* and *in vivo* passage. Also, Lacaze and colleagues [13] extended the microarray approach to include probes specific to both strands of the genome, leading to the discovery of noncoding and bi-directional transcription at late stages of MCMV infection. Finally, a recent transcriptomic analysis of newly synthesized RNA in MCMV infected fibroblasts [15] applied RNA-Seq technology to study regulation of viral gene expression and identified a very early peak of viral gene transcriptional activity at 1–2 hours post infection followed by rapid cellular countermeasures but did not attempt to re-annotate MCMV genome.

Altogether, these new technologies have refined and advanced our knowledge of viral genes and the MCMV genome. Nevertheless, we still lack definitive annotation for the standard lab strains of MCMV and specific knowledge of how many of these genes function during natural infection and disease. Currently, two annotations of MCMV genomes are used – the original Rawlinson's annotation with minor modifications (GenBank accession no. GU305914.1) where 170 open reading frames (ORFs) are identified and the NCBI reference sequence annotation (GenBank accession no: NC_004065.1) with 160 ORFs. We previously used a transcriptomic approach to analyze gene products of HCMV [16]. This was the first report to characterize abundant antisense and noncoding transcription in the HCMV genome showing that there is greater complexity of herpesvirus genomes than previously appreciated. Using RNA-Seq technology, Gatherer *et al.* [17] showed that most protein coding genes are also transcribed in antisense but are generally expressed at lower levels than their sense counterparts. A more recent analysis of translational products of HCMV [18] by ribosomal footprinting indentified 751 translated ORFs, further underscoring the complexity of herpes virus genomes.

We describe MCMV transcriptional products that differ from predicted ORFs, novel spliced transcripts, and novel transcripts derived from intergenic regions of the genome. Additionally, we found that the most abundant viral transcript (MAT) is a spliced transcript recently identified as a noncoding RNA that limits accumulation of cellular miRNAs [19], [20]. Here we report that this transcript also specifies a novel protein and to our knowledge, this is the first viral transcript that functions both as a noncoding RNA and mRNA. Analysis of the host transcriptional response to infection revealed many unexpected host genes that are regulated by virus infection, including many noncoding RNA genes. Correspondingly, many host genes regulated by virus infection cluster in unexpected biological pathways that may shed new light on the pathogenesis of cytomegalovirus-associated diseases. Together, these findings suggest important revisions are required for MCMV genome annotation and emphasize numerous aspects of MCMV biology and the host response to this infection that are unknown and require further study.

## Results

### The MCMV transcriptome

In this study, we set out to complete a transcriptomic analysis of MCMV infection. We analyzed viral transcripts through classical cDNA cloning and sequencing and through next generation sequencing of cDNA generated from total cellular RNA (RNA-Seq). Analysis of cDNA libraries is a well-proven approach to analyze viral transcripts based on isolation of long transcripts, molecular cloning of the transcripts, and traditional Sanger-based sequencing of the cDNA clones. Traditional cloning has many advantages, including isolation of novel transcripts that may not be identified by probe-based technologies, as well as precise analysis of transcript splice sites and transcript 3′ ends. The introduction of massively parallel sequencing techniques represents a major new technology to study gene expression. Basically, RNA (total or fractionated) is converted to a library of smaller cDNA fragments. Adaptors are added to the fragments, and the shorter fragments are sequenced in a high-throughput manner using next generation sequencing technology. This RNA-sequencing (RNA-Seq) approach is free of selection biases associated with traditional cloning or probe-based methods and allows for the entire transcriptome to be analyzed in a quantitative manner (reviewed in [21]).

First, cDNA libraries representing the major temporal classes of viral gene expression were generated by collecting RNA from infected mouse embryonic fibroblasts (MEFs) at 9 time points after infection. For RNA-Seq analysis, RNAs collected at the same 9 time points were pooled, converted to cDNA, and sequenced on the Illumina Genome Analyzer IIx. Of the 33,995,400 reads that passed the filter from infected cells, 11% aligned to MCMV genome indicating a 585-fold coverage of the viral genome.

A total of 448 cDNA clones were included in the final analyses [84 from the immediate early (IE) library, 163 from the early (E) library, and 201 from the late (L) library]. Generally, temporal assignment of cDNA clones in this study agrees with previous studies and a detailed comparison, including discrepancies to earlier studies is provided in **Dataset S1**.

**Figure 1:**
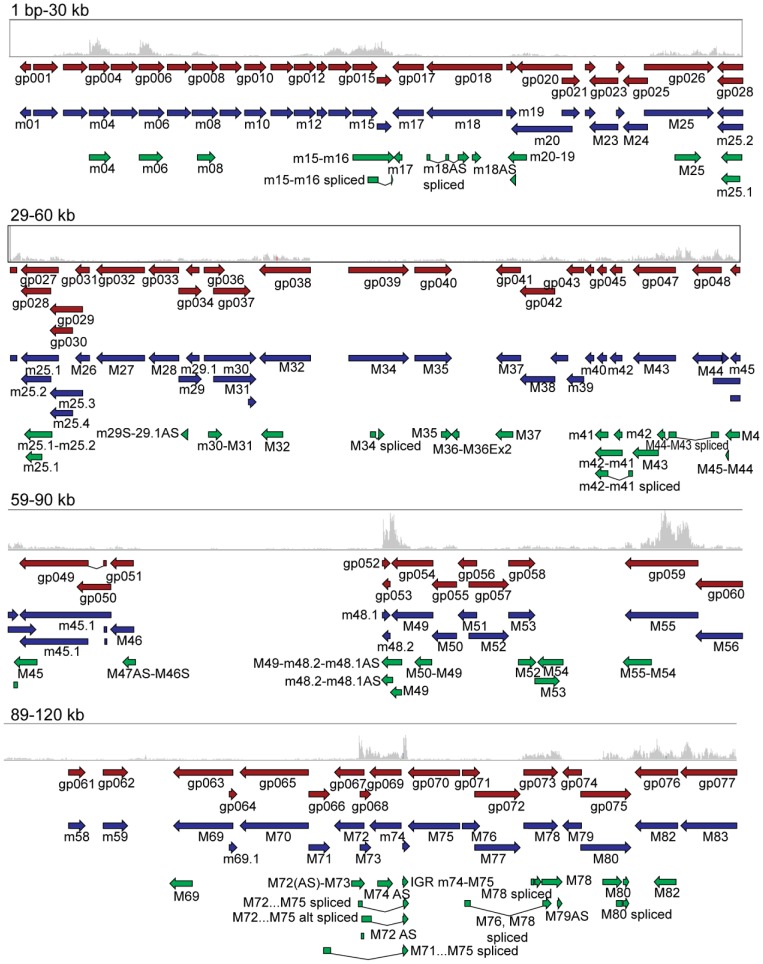
Comparison of cDNA cloning and RNA-Seq data in relation to current genome annotation. Comparison of poly(A) cDNA library (green arrows) and RNA-Seq analysis of murine cytomegalovirus (gray histograms). The longest clone from each group of clones in the cDNA library is shown. ELAND alignments of RNA-Seq reads were loaded in Integrative Genomics Viewer and compared to NC_004065.1, (red arrows) and GU305914.1 (blue arrows). The data range for RNA-Seq data was set to 20–5000. Data is shown in 30 kb ranges with 1 kb overlap. Data is shown for the first 120 kb of the MCMV genome and the figure legend is shown in Figure 2.

As shown in **Figures 1** **and** **2**, transcriptomic data generated using these two experimental approaches were compared to currently available genome annotation (the NCBI reference sequence, GenBank accession. no. NC_004065.1, and a more recent sequence analysis of the Smith strain, GenBank accession no. GU305914.1). Using this schematic overview, current annotations (red and blue arrows) largely agree. The MCMV transcripts identified through our classical cDNA cloning and sequencing (green arrows) and the RNA-Seq expression profiles (gray histograms), showed complementary results to each other but diverged dramatically from current annotations. A summary of the cDNA clones relative to genes annotated in the NCBI reference sequence (NC_004065.1) is shown in **Table S1**, and a complete list of the 448 cDNA clones isolated in this study and their characteristics are presented in **Table S2**. A detailed comparison of transcripts cloned in this study compared to current annotations (GU305914.1 and NC_004065) is presented in **Table S3**, and includes estimated relative expression measured as reads per kilobase per million mapped reads (RPKM) derived from RNA-Seq analysis.

**Figure 2:**
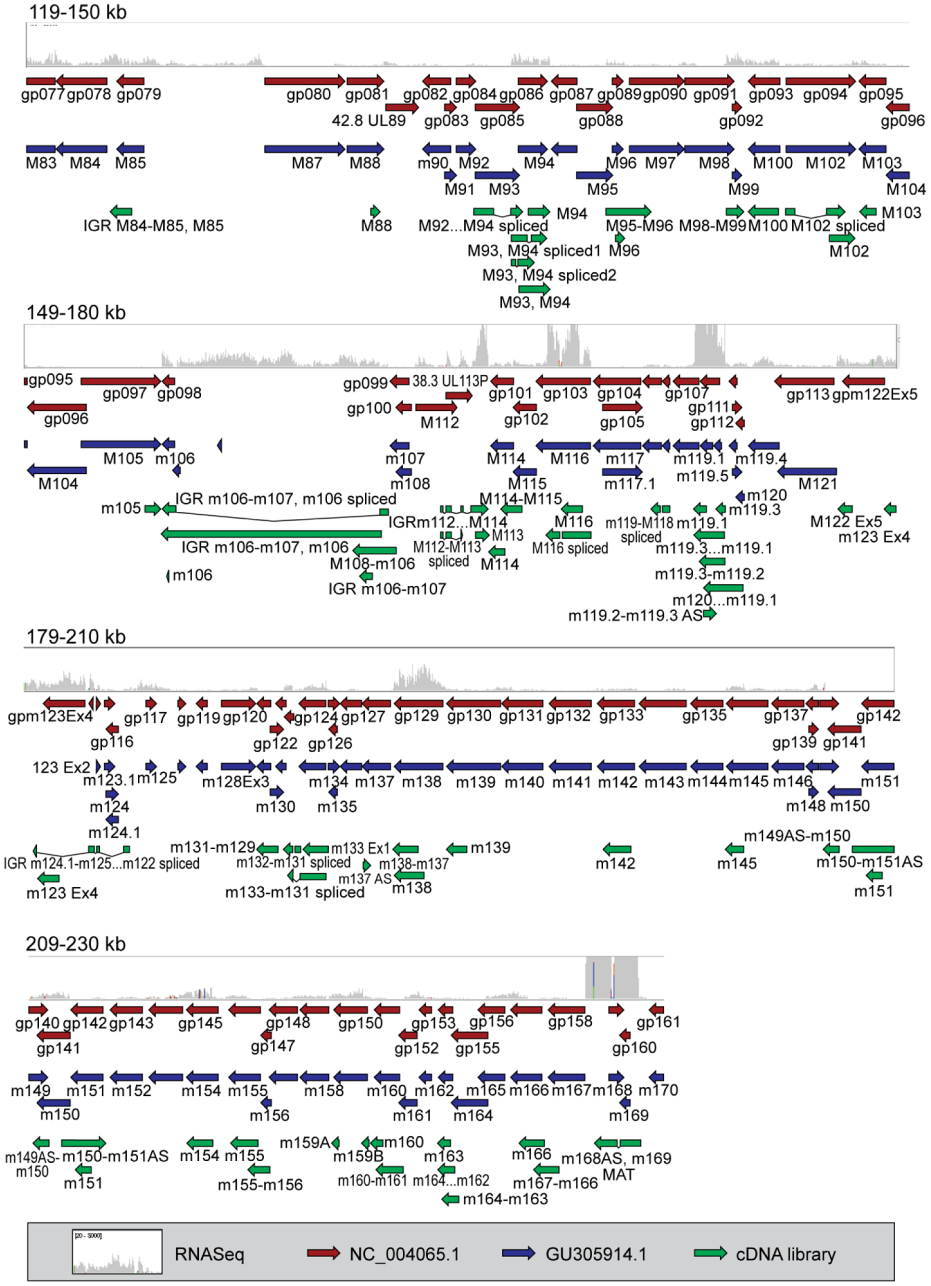
Comparison of cDNA cloning and RNA-Seq data in relation to current genome annotation. Comparison of poly(A) cDNA library (green arrows) and RNA-Seq analysis of murine cytomegalovirus (gray histograms). The longest clone from each group of clones in the cDNA library is shown. ELAND alignments of RNA-Seq reads were loaded in Integrative Genomics Viewer and compared to NC_004065.1, (red arrows) and GU305914.1 (blue arrows). The data range for RNA-Seq data was set to 20–5000. Data is shown in 30 kb ranges with 1 kb overlap. Data is shown for genomic region spanning 119–230 kB of the MCMV genome.

Analysis of the cDNA clones revealed many novel transcripts with the following characteristics:

1. **Novel antisense transcripts.** Excluding 20 cDNA clones that mapped to intergenic regions, 275 (64%) of cDNA clones were in the sense (S) orientation, 39 (9%) were antisense (AS), and 114 (27%) overlapped more than one gene in both S and AS orientations relative to original annotation provided by Rawlinson and colleagues [9]. These designations were re-evaluated according to the current NCBI reference sequence (NC_004065) in which AS transcripts to hypothetical or putative proteins were revised as S transcripts due to lack of evidence for the predicted sense transcript. If AS transcripts that overlapped hypothetical proteins also overlapped S transcripts in this library, the AS designation was preserved. According to these criteria, 431 (97%) transcripts were in S orientation, only 4 (0.09%) were in AS orientation, and 9 (2%) overlapped more than one gene in both S and AS orientations.
2. **Transcripts overlapping more than one currently annotated genes.** Several cDNA clones were isolated that overlap more than one currently annotated gene. For example, four cDNA clones in our library overlapped both the m15 and m16 genes. The longest of these clones specifies a 1673 bp transcript, whereas current annotation predicts two genes of 908 bp (*m15*) and 632 bp (*m16*). These data suggest the possibility that several ORFs are translated from unexpectedly long or polycistronic transcripts. Unusually long and polycistronic transcripts have recently been shown to be a feature of HCMV transcriptome [18].
3. **The absence of transcripts in currently annotated regions.** Several gene regions were not well represented in the cDNA cloning study. For example, no cDNA clones overlapping m01 or m170 were found in this study, and these regions had the lowest RPKM reads by RNA-Seq of 132 and 141, respectively (gray histograms in **Figures 1** and **2** and **Table S3**), consistent with earlier gene array-based studies [12]. For comparison, the well-defined MCMV genes *m04* and *m138*, both represented with multiple clones in our cDNA library, have RPKM values of 14,137 and 16,935 respectively. Because some viral transcripts with higher RPKM reads were not represented in the classical cDNA library (for example, an RPKM of 2967 for the *M98* gene), we conclude the cDNA library cloning did not capture all viral transcripts. Nevertheless, the failure to identify clones in regions poorly represented by RNA-Seq data suggests that further attention is required to prove or disprove the existence of genes predicted by *in silico* (ORF) analysis.
4. **Novel spliced transcripts.** One of the most striking aspects of the cloning study was the abundance of novel spliced transcripts. A total of 22 novel spliced transcripts were cloned in this study as well as spliced transcripts reported by others. A complete list of spliced transcripts identified in this and other studies is provided in **Table S4**. One of the most abundant novel spliced transcripts identified in this study maps to the *M116* gene (8 cDNA clones). Current annotation for M116 predicts a protein of 645 AA, whereas the clones in this study predict a novel truncated protein product. Splicing results in a change of open reading frame at amino acid residue 350 of the currently annotated M116 protein and introduction of a new stop codon at position 401 (**Dataset S2**). By far the most abundant novel spliced transcript is that overlapping *m169* in the S orientation, and *m168* in the AS orientation and is discussed in detail below.

### The most abundant MCMV transcript (MAT)

**Figure 3:**
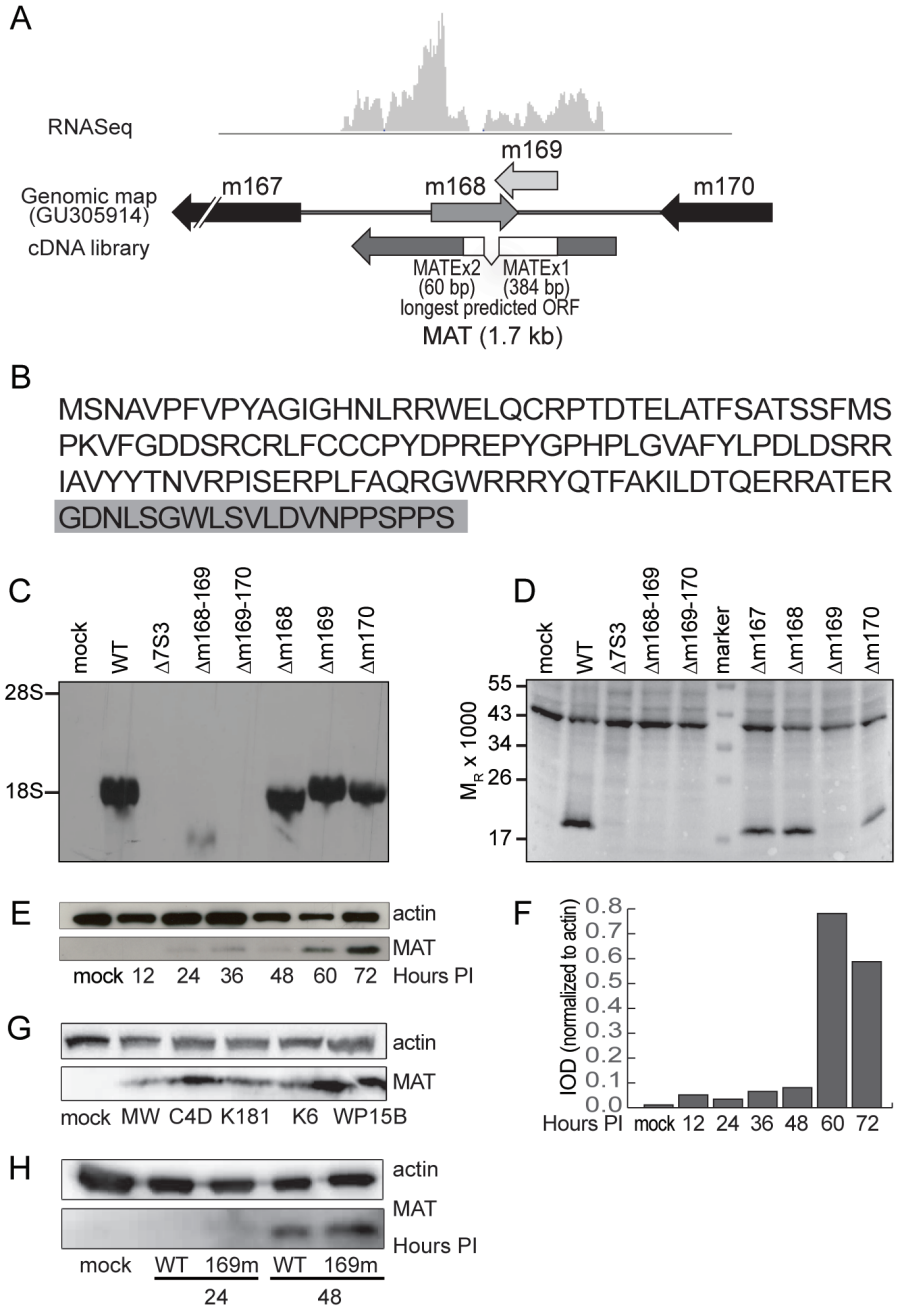
Analysis of the novel most abundant MCMV transcript and protein. (A) Comparison of RNA-Seq data and the longest MAT cDNA clone (E125) with current annotation (GU305914). The predicted exons are shown in white boxes. (B) Predicted amino acid sequence of the MAT protein. The first 127 residues match a truncated m169 translation and the C-terminal 20 residues highlighted in gray are derived from exon 2, mapping to the m168 gene. (C) Northern analysis of MAT RNA in MEF cells infected with various deletion mutants. Note that the single gene mutants are partial gene deletions and thus truncated transcripts accumulate. (D) Immunoblot analysis of MEF cell lysates probed with monoclonal antibody generated to the predicted m169 ORF or monoclonal antibody to actin (45 kDa band). (E) Immunoblot analysis of the time course of MAT protein accumulation in infected cells and (F) quantitation. (G) Immunoblot analysis of MAT protein from cells exposed to wild virus isolates. (H) MAT protein accumulation in WT and m168mut virus infected Balb/c MEF. Mutation of the binding site for miR27-b in MAT 3′UTR did not alter regulation of MAT protein expression.

Approximately 31% of the viral cDNA clones and 41% of all viral reads from the RNA-Seq analysis mapped to the novel spliced transcript at the right end of the genome. The structure of this transcript relative to current gene annotation is shown in **Figure 3A** and the spliced nature of this transcript is also apparent in the RNA-Seq profile (gray histogram). The longest predicted ORF extends into the second exon and predicts a protein of 147 AA, of which the first 127 residues matches the predicted m169 protein sequence (**Figure 3B**). To determine if this transcript is translated, an antibody was prepared to the protein sequence predicted for *m169*. This antibody was used in immunoblot analysis of cell lysates prepared from mock-infected cells and cells infected with wild-type (WT) BAC-derived Smith strain virus, or various multi-gene and single-gene mutant virus strains. This antibody reacted with a protein of approximately the expected size (17 kDa) (**Figure 3D**) in cells infected with WT virus and mutant viruses that express most or all or the MAT transcript as determined by northern blot analysis (**Figure 3C**).

While the MAT protein is first detected 24 hrs after infection, maximal amounts are observed at 60 and 72 hrs after infection (**Figure 3E**). MAT protein was also detected in fibroblasts exposed to five different strains of MCMV isolated from wild mice, indicating that the coding region of the gene is conserved in laboratory and wild strains of MCMV (**Figure 3G**). Most remarkably, these findings demonstrate that the MAT gene generates a single transcript with both noncoding [19], [20] and protein coding functions.

We also investigated the possibility that MAT protein accumulation is directly related to control of the MAT transcript levels by cellular miR-27 [20]. Marcinowski and colleagues have shown that when the binding site for miR-27 is mutated (m169-mut virus), MAT transcript levels were increased twofold in comparison to levels obtained in cells infected with WT MCMV at 24 hours after infection due to loss of transcript regulation by the microRNA. The difference in MAT transcript abundance between WT and the m169-mut virus was lost by 48 hours after infection. However, we observed that MAT protein levels were similar in cells infected with the WT and m169-mut viruses at 24 and 48 hrs after infection (**Figure 3H**). We conclude that the noncoding function of the MAT transcript (regulation of cellular miR-27) is unrelated to MAT protein accumulation.

### RNA-Seq analysis of viral gene expression

**Figure 4:**
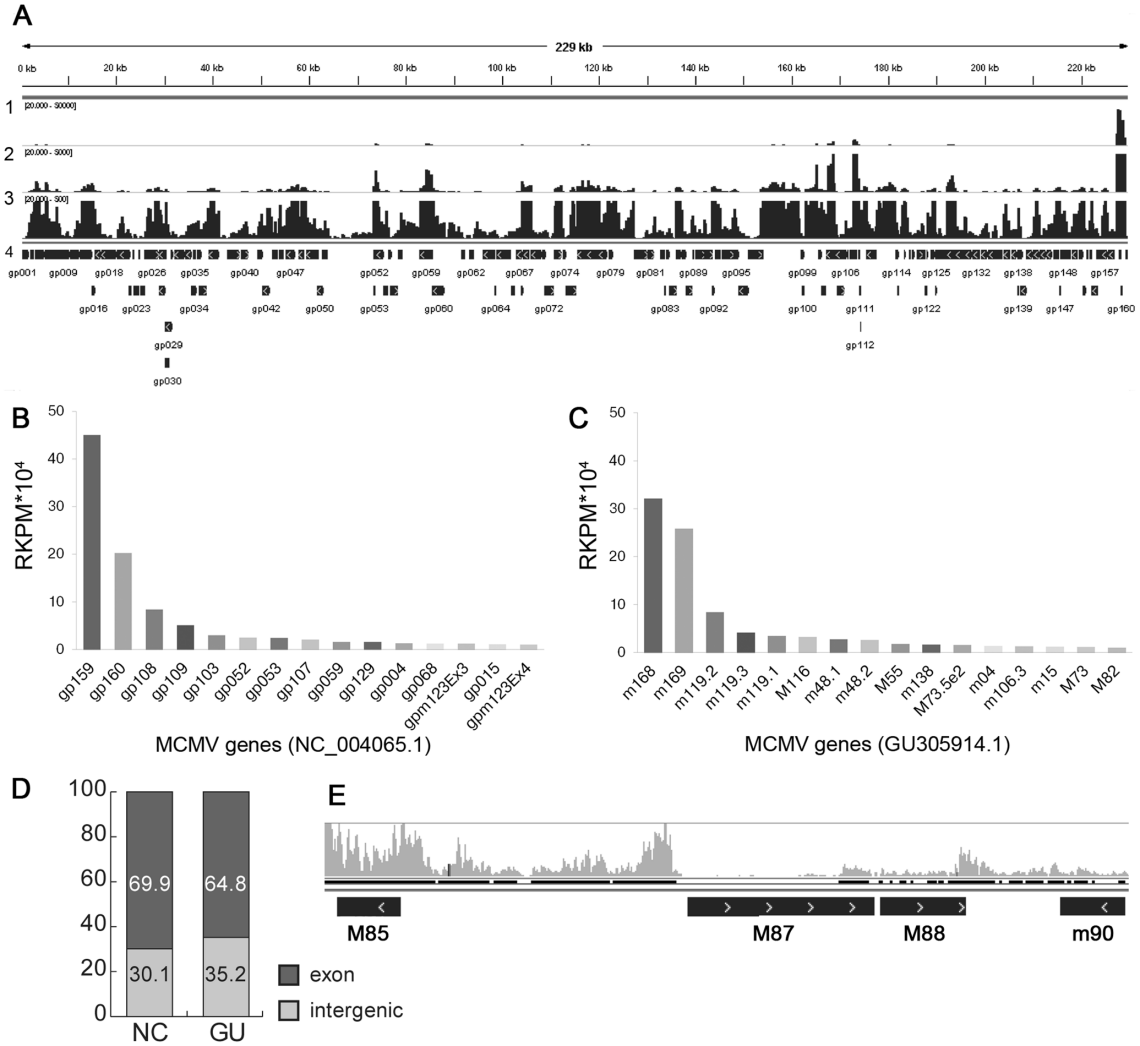
Transcriptional activity of MCMV. (A) Whole genome visualization using IGV viewer of RNA-Seq reads mapping to the MCMV genome showing different data ranges. Row 1, range of 20–50,000 reads; Row 2, range of 20–5000 reads; Row 3, range 20–500 reads; Row 4, annotation from NC_004065. (B to D) Quantitation of transcript abundance varies with annotation. The most expressed MCMV genes (RPKM>10 000) relative to NCBI NC_004065 and (B) and GU305914.1 (C). (D) Percentage of reads mapping to coding (exon) or intergenic regions using NC_004065.1 (NC) or GU305914.1 (GU). (E) Example of a transcriptionally active region between M85 and M87.

In addition to providing valuable insight into transcript structure, RNA-Seq analysis revealed several new facets of the viral transcriptome. First, accumulation of individual viral transcripts varies by several orders of magnitude. **Figure 4A** depicts the number of RNA-Seq reads mapped against the MCMV genome in which rows 1, 2 and 3 visualize the data with the maximum number reads set to 50,000, 5000, and 500, respectively. Validating the classical cloning study, the most abundant transcript identified by RNA-Seq is the MAT transcript. Enumeration (RPKM) of the most abundant transcripts is presented in **Figures 4B and 4C**, and shows that after the MAT transcript, the most highly expressed genes are *m119, M116*, and *m48*, all genes without assigned functions. Also highly expressed are the immune evasion genes *m04* and *m138, M55* (glycoprotein B), and additional genes of unknown functions (*M73* and *m15*). Second, as shown by comparing **Figure 4B** to **C**, both the overall magnitude of expression and the ranking of the abundance of different transcripts vary according to annotation used for the RPKM analysis. Third, analysis of reads mapped at the highest resolution (**Figure 4A**, row 3) indicates that most of the viral genome is transcribed to some degree. Remarkably, 30 or 35% of the reads mapped to intergenic regions, depending on annotation (**Figure 4D**). This percentage is reduced to 14% when annotation is modified to reflect the correct MAT gene structure identified in this study. RNA-Seq also detected significant transcription in *m74-M75* (**Figure S4**), *M85-M87* and *M88-m90* (**Figure 4E**) intergenic regions. In contrast, the transcriptional profile of the annotated M87 shows less transcriptional activity than the adjacent intergenic regions. Similarly, RNA-Seq identified transcription from genes that were not isolated in the classical cDNA library or in previous studies using microarray technology [12], [13]. A detailed analysis of the sensitivity of this RNA-Seq study to previous studies is provided in **Supplemental Datasets S1A–C**. We also compared our RNA-Seq data to a recent RNA-Seq analysis of the MCMV transcriptome using BAC-derived WT virus on NIH-3T3 fibroblasts [15]. As shown in **Supplemental Figure S1**, the profiles obtained from these two different RNA-Seq experiments are remarkably similar despite using different sequencing platforms and library generation approaches. Also, either seven or eight of the 10 most abundant genes were identical in both datasets (**Supplemental Dataset S1C**). Minor differences in abundance of some transcripts can be attributed to differences in the time points analyzed in these two studies as well as the fact that our analysis achieved an order of magnitude greater sequencing depth (compare reads analyzed for each histogram set in **Figure** S1).

Together these findings demonstrate that RNA-Seq analysis is a highly sensitive method for detection of viral gene expression during infection. Moreover, these findings highlight numerous incongruencies with current annotation for the MCMV genome. Finally, RNA-Seq analysis revealed that many of the most abundantly expressed viral genes are of unknown function.

### Northern analyses of novel transcripts

**Figure 5:**
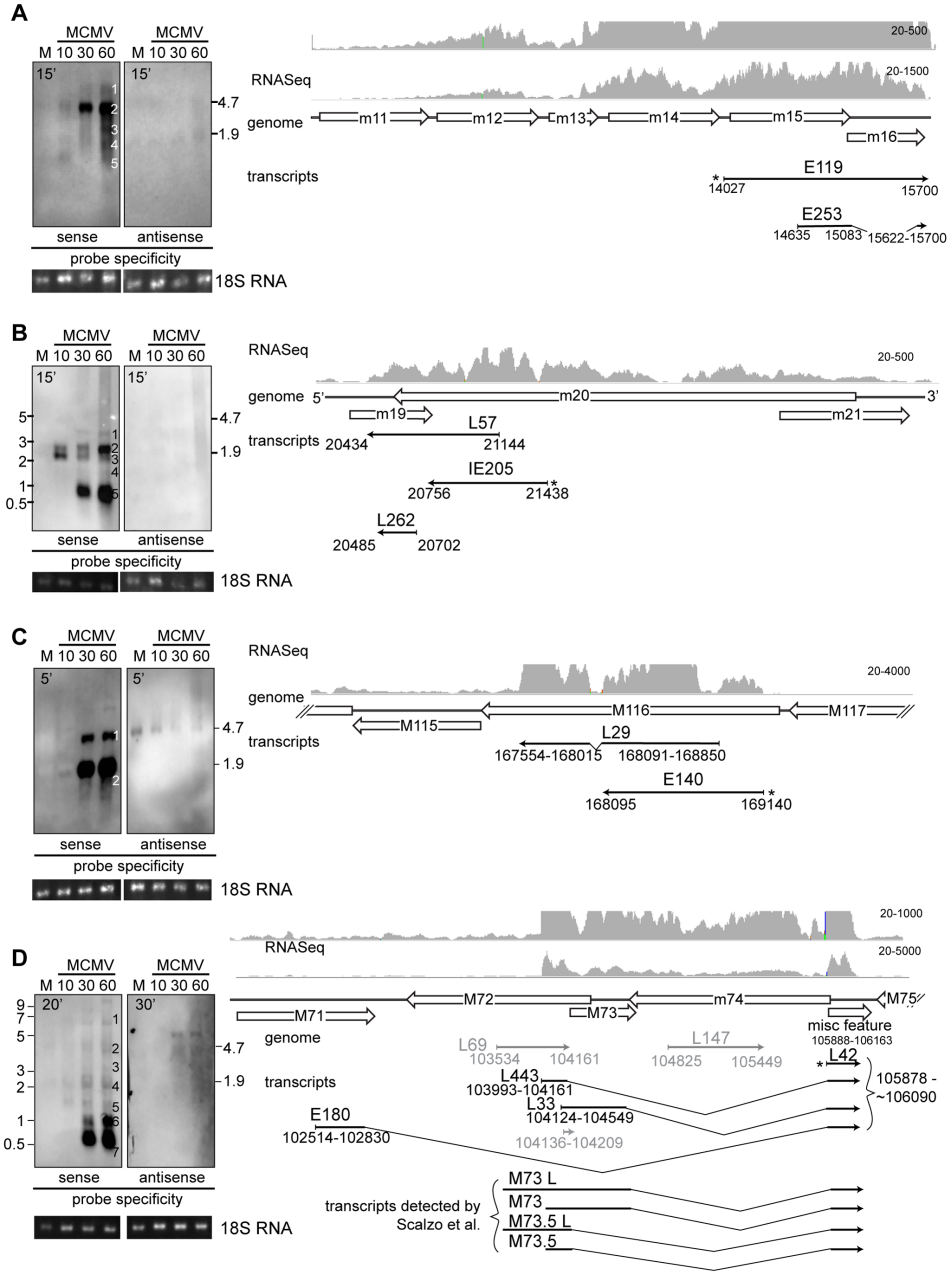
Verification of new transcripts by northern blot. Balb/c MEF cells were infected with BAC derived Smith virus and harvested at indicated times post infection. Total RNA was separated by denaturing gel electrophoresis, transferred to nylon membrane and incubated with probes specific for S and AS transcripts. RNA integrity and loading was evaluated by inspecting 28S (not shown) and 18S rRNA bands under UV light after transfer to membrane. Transcripts in the *m15–16* (A), *m19-m20* (B), *M116* (C) and *M71-m74* (D) gene regions were analyzed (Due to smiling effects during gel electrophoresis for the image shown in 4A and C, the ladder was not accurate for inner lanes of the gel and the position of the ribosomal bands was therefore used to estimate the band sizes). Predicted genes (Rawlinson's annotation) are depicted as empty arrows, while thin black arrows show longest transcripts cloned in our cDNA library as well as clones used to generate probes (marked with *). 3′ ends of transcripts are marked with arrowheads. The nucleotide coordinates relative to Smith sequence (NC_004065.1) of isolated transcripts are given below thin arrows, while the names of the clones are written above. Thin gray lines show isolated transcripts that cannot be detected with the probe. Gray histograms showRNA-Seqreads aligned to MCMV genome. Maximal possible exposure times were used to ensure even low abundance transcripts are detected and are noted on the blots.

Because cDNA cloning and RNA-Seq identified significant differences between the MCMV transcriptome and current annotations, we performed an in depth analysis of several genomic regions by northern analyses (**Figure 5**, **Figures S2, S3, S4, S5**) using our cDNA clones to generate strand specific riboprobes (**Table 1**).

**Table 1:** cDNA clones and PCR primers used to generate probes for Northern analyses.

	antisense probe			sense probe
Region	clone name	genomic location	genomic strand	PCR primers*
m15–m16	E119	14027–15700	+	F: AATTAACCCTCACTAAAGGGAAAAGTATTGCGTATAAGACACT
				R: TCAAGAAGATGTACCGTCAC
m20–19	IE205	21144-20434	−	F: AATTAACCCTCACTAAAGGGAGAAAAGATTCTTTATTGCGTCGAG
				R: AGCGCGATGCTGTTACG
m20–19	L57	21371-20436	−	F: NA
				R:NA
m72	L69	103534–104161	+	F: AATTAACCCTCACTAAAGGGGCTCCGGTCCGCCCGAAT
				R: GGCAGCTCCAGCGGACCC
m74	L147	104825–105449	+	F: AATTAACCCTCACTAAAGGGACAGAGGTGGCGAGCATCAAA
				R: GAAAAATTGTATCGGGTGCATGTTTTC
M75	L42	105878–106095	+	F: AATTAACCCTCACTAAAGGGAGAAAAGATTCTTTATTGCGTCGAG
				R: AGCGCGATGCTGTTACG
M100	E126	145353-144169	−	F: AATTAACCCTCACTAAAGGGCGCGTATCTCTTCGTTGTCCA
				R:ATTACCCGCGCATCATCGAC
M102	E14	147457–148161	+	F: AATTAACCCTCACTAAAGGGTCGTCTTTTGCAGTGTGTCT
				R: CATCCGCTTCATGGCCAC
M103	L51	148772-148169	−	F: AATTAACCCTCACTAAAGGGTTTTATTGTTCGAGGCGCTTT
				R: ACCTTCCTGACCGGCACCA
M116	E140	169140-168095	−	F: AATTAACCCTCACTAAAGGGCCTGCTGAGGAGTAGTCTTGG
				R: TGTCGGCGCGCTGCTCT

* T3 promoter sequences are underlined.

To investigate genomic regions where transcripts overlapping more than one gene were detected, we analyzed transcription in m15–16 and m19–20 regions. In both regions multiple transcripts were detected with different temporal expression patterns. Smaller transcripts tended to accumulate at later time points, a feature previously reported for certain transcripts in both HCMV and MCMV [22]–[24]. In the m15–m16 region 5 transcripts were cloned, all of which overlapped the predicted m15 and m16 genes, and one transcript was spliced (**Figure 5A**). The RNA-Seq profile (**Figure 5A**, gray histogram) also strongly indicated transcription that spans both predicted genes. Consistent with our cDNA library, no antisense transcripts were detected while the sense probe detected 5 transcripts (**Figure 5A**, bands 1–5). The 3′ end of all cDNA clones end at or close to nucleotide position 15700 (**Supplemental Table S2**) and RNA-Seq data alignment to MCMV genome indicates a sudden drop in reads around this nucleotide position. Assuming that transcripts in this region are co-terminal, band sizes predict transcript initiation sites in *m11, m12, m13 or m14, and m15* (**Figure 5A**, bands 1–4). Similar results were found in this region in cells infected with wild isolates of MCMV (Alec Redwood, personal communication). While the smallest band observed by northern analysis (**Figure 5A**, band 5) corresponds in size to the novel spliced transcript, E253 (566 bp), we could not confirm splicing by PCR using intron-flanking primers (data not shown). Therefore, it is likely this spliced transcript represents an aberrant transcript or a result of intramolecular template switching during reverse transcription [25].

The *m20* gene region also diverged substantially from current annotation. Similar to *m15–16* region, in the *m20* region 5 transcripts with differential temporal expression patterns were detected by northern analyses using clone IE205 as a probe (**Figure 5B**). No transcript was detected using an AS probe derived from clone IE205 or L57, indicating that *m19* is not transcribed in the sense orientation (**Figure 5B** and **Figure S2**). We therefore propose that *m19* should be removed from MCMV genome annotation. This is consistent with our cloning study where 4 transcripts have been isolated in this region and none overlap *m19* in the sense orientation (**Supplemental Table S2**). Evidence from the cDNA library and RNA-Seq alignment (**Table S2** and **Figure 5B**) indicates that 5 bands detected in northern are co-terminal, with the 3′ end located close to nucleotide position 20430. The largest band at 4 kb (**Figure 5B**, band 1) is detectable at 30 and 60 hours PI and we predict that this transcript initiates in *M23*. We failed to detect transcripts between the *m20* and *m25* genes consistent with previous studies [12] and northern analyses using mutant viruses lacking genes in this region (data not shown). The lack of cDNA clones can be explained by low abundance and size of this transcript, as well as by propensity of cDNA libraries to enrich 3′ ends. The band slightly smaller than 3 kb (**Figure 5B**, band 2) shows a peak accumulation at 60 hours PI and is consistent with a transcript overlapping *m19-m21* (approx locations 20430–23220, 2.79 kb). The band of approximately 2 kb (**Figure 5B**, band 3) shows peak accumulation 10 hours PI. Based on the RNA-Seq profile this band could represent transcripts that initiate at nucleotide position 22060. Finally, the late time points are dominated by smaller transcripts of approximately 1 kb (**Figure 5B**, band 5; predicted start site at 21440) which correspond in size to the cDNA clones detected in our study. In short, northern analyses support the conclusion that transcripts overlapping multiple genes in the *m15-m16* region and *m19-m20* region accumulate in infected cells and indicate that additional, larger transcripts are transcribed in these regions which have yet to be characterized.

Next we analyzed gene regions in which we detected novel spliced transcripts. The *M116* region was chosen as an example of a highly abundant spliced transcript of unknown function in addition to m168–169 transcript. Current annotations predict an ORF of 1.9 kb whereas RNA-Seq profiles and cDNA study both detected a slightly shorter (1.6 kb) transcript with an 81 bp intron. Northern blot analysis (**Figure 5C**) identified a strong band of appropriate size (1.6 kb) that starts to accumulate at IE times and peaks at E and L times after infection. Due to the small intron and high abundance of this transcript, unspliced transcripts could not be definitively resolved by northern analysis but were confirmed by PCR using primers flanking the intron (**Figure S3**). Additionally, northern analysis detected another less abundant band of approximately 3 kb. Leatham and colleagues [26] have detected a band of similar size in the homologous region in HCMV of 3.2 kb that encompasses *UL119-115* genes. While we failed to isolate cDNA clones overlapping *m117* region, we predict the larger, less abundant 3 kb band observed initiates in *m117*, though additional northern or 5′RACE studies are needed to confirm the start site of the larger transcript.

The *m72-m74* region was previously shown to have a very complex transcriptional profile [23], [27]. cDNA library data, RNA-Seq data and results of northern analyses with L42 as a probe all are in agreement with the findings of Scalzo et al. [23] of multiple spliced transcripts that share exon 2. Bands 5–7 (**Figure 5D**) correspond to previously reported *m60*, *m73* and *M73.5* spliced transcripts. In our cloning study four isolated clones correspond to *M73.5* transcripts (represented by the longest clone, L443) and one to *M73* (L33) (**Supplemental Table S4**). Transcripts corresponding to *m60* were not isolated in the cloning study, however the RNA-Seq profile in the region corresponding to *m60* exon1 shows active transcription (**Figure S4D**). We have also detected a 1.1 kb band (**Figure 5D**, band 6) that corresponds to longer *M73* and *M75* transcripts, and bands corresponding to unspliced transcripts of *M73* and *M73.5* (approx. 2 kb, **Figure 5D**, band 4). In addition to these previously published transcripts, we have also cloned one novel spliced transcript from this region, E180. In accordance with work in the *m60* region [23], we propose *M71S* as a designation for this novel gene. Like other spliced transcripts from this region, E180 shares exon 2 with other transcripts while its splice donor site is located at 102830. The spliced nature of this transcript has been confirmed by PCR (**Figure S4C**), however more analyses are needed to determine its exact 5′ start site. Northern analysis revealed a band of 0.5 kb (**Figure 5D**, band 7) that corresponds in size to the E180 spliced transcript whereas the unspliced version is detected around 3 kb (**Figure 5D**, band 3). In addition, a band of similar size (3.5 kb) transcribed from the plus genomic strand detected by the L147 probe (**Figure Figure S4B**) could correspond to the unspliced version of E180. All probes used in this region detected bands transcribed from negative genomic strand that correspond to those previously reported by Rapp et al. [27]. Based on additional northern blots using cDNA clones L69 (AS to m72) and L147 (AS to m74) **(Figure S4A and B**) as well as previous reports [23], [27] we conclude that the 5 kb transcript starts in *m75* and ends in *m72* and corresponds to transcript encoding gH while the 3 kb transcript starts in *m74* and ends in *m72* and corresponds to transcript encoding dUTPase (**Supplemental Figure S4**). Additional very large transcripts transcribed from plus or minus genomic strands detected by the L42 and L147 probes, respectively, have yet to be characterized but underscore the complex transcriptional patterns in this region.

Last, we set out to confirm novel antisense transcripts detected in the cDNA library. Analysis of transcription in *M100-M103* region confirmed previously published findings. We have detected single M102 transcript from plus genomic strand as described by Scalzo [28] (**Figure S5A**). A probe derived from *M100* detected a single transcript from negative genomic strand corresponding to M100 [28], and 2 from the positive genomic strand that correspond to those described by Cranmer et al. [29] and are in line with our cDNA and RNA-Seq analysis (**Figure S5B**). The presence of sense and antisense transcripts in this gene region corresponds to findings for HCMV [16]. Finally, in the *M103* gene region we detect 2 transcripts from plus genomic strand that correspond to those described by Lyons et al [30] (**Figure S5C**). Temporal expression of transcripts detected by northern in this region is in line with our cDNA analysis and previously published data [13], [15].

Based on northern analyses of 5 regions, we conclude that our cDNA and RNA-Seq analyses faithfully represents the MCMV transcriptome in infected primary fibroblasts and confirms the presence of novel transcripts. Moreover, the distribution of clones in the IE, E and L cDNA libraries accurately reflected the accumulation of transcripts detected by northern analyses.

### The host cell response to MCMV infection

RNA-Seq analysis also enabled us to investigate changes in the host transcriptome. Differentially expressed (DE) murine genes in MCMV-infected cells compared to mock-infected cells were identified by calculating RPKM. This analysis identified 10,748 statistically significant (p<0.05) genes altered by infection (**Table S5**). The top induced, upregulated, repressed and downregulated genes are presented in **Tables 2** **–** **5**, (genes associated with characterized biological pathways are in bold). *Interferon β (Ifnb1*) and the interferon-inducible *pyhin1* (a.k.a. *ifi-209*, *ifix*) were among the top induced genes, consistent with the expected host response to virus infection. Also congruous with expected host responses to infection were two highly induced genes associated with apoptosis induction (*Hrk* and *Tnfsf10* [a.k.a. TRAIL]). Interestingly, transcription factors (*Foxa1, En2, Insm1*, *Tbx21*, [a.k.a *T-bet*], and *Tp73*) were among the most strongly induced by MCMV infection.

**Table 2:** Top 20 host genes^1^ induced in infection.

Gene	Full name	Fold change
Ankrd34b	ankyrin repeat domain 34B	34.4
**Ifnb1**	**interferon beta 1**	**34.1**
**Foxa1**	**forkhead box A1**	**34.1**
Spint1	serine protease inhibitor, Kunitz type 1	33.8
Lin28b	lin-28 homolog B	33.8
**En2**	**homeobox protein engrailed-2**	**33.3**
**Hrk**	**harakiri, BCL2 interacting protein (contains only BH3 domain)**	**33.0**
**Insm1**	**insulinoma-associated 1**	**33.0**
**Pyhin1**	**pyrin and HIN domain family, member 1; ifi-209; interferon-inducible protein 209**	**32.9**
**Tnfsf10**	**tumor necrosis factor (ligand) superfamily, member 10**	**32.9**
**Gabrq**	**gamma-aminobutyric acid (GABA) A receptor, subunit theta**	**32.7**
1110032F04Rik	RIKEN cDNA 1110032F04 gene	32.6
Cnpy1	canopy 1 homolog	32.5
Slc35d3	solute carrier family 35, member D3; Frcl1	32.4
Esx1	extraembryonic, spermatogenesis, homeobox 1; Spx1	32.4
Esrp1	epithelial splicing regulatory protein 1; Rbm35a	32.3
**Tbx21**	T-box transcription factor TBX21, T-bet	**32.3**
Trim71	tripartite motif-containing 71; Lin41	32.2
**Trp73**	transformation related protein 73	**32.0**
Cpne5	copine V; A830083G22Rik	32.0
**Cdh7**	**cadherin-7**	**32.0**

1 p<0.05 identified using SAMMate with EdgeR.

Genes associated with genetic networks identified by IPA are shown in bold.

**Table 3:** Top 20 host genes^1^ upregulated in infection.

Gene	Full name	Fold change
Art3^2^	ADP-ribosyltransferase 3	8.7
**Cxcl10**	**chemokine (C-X-C motif) ligand 10**	**8.5**
**Ccl5**	**chemokine (C-C motif) ligand 5, RANTES**	**8.5**
Trank1	tetratricopeptide repeat and ankyrin repeat containing 1	8.4
**Cxcl9**	**chemokine (C-X-C motif) ligand 9; Mig**	**8.1**
**Rsad2**	**radical S-adenosyl methionine domain containing 2; virus inhibitory protein**	**7.9**
Dsg2	desmoglein 2	7.9
**Mx1**	**myxovirus (influenza virus) resistance 1**	**7.1**
**Ugt8**	**UDP galactosyltransferase 8A**	**7.0**
**Cxcl11**	**chemokine (C-X-C motif) ligand 11; interferon-inducible T-cell alpha chemoattractant**	**6.9**
Tex16	testis expressed gene 16	6.9
**Gpr50**	G protein-coupled receptor 50; melatonin-related receptor	**6.8**
**Jag2**	Jagged2	**6.7**
**Oasl1**	2′-5′ oligoadenylate synthetase-like 2	**6.5**
Cited1	Cbp/p300-interacting transactivator with Glu/Asp-rich carboxy-terminal domain 1; Msg1	6.5
Kcnq2	potassium voltage-gated channel, subfamily Q, member 2	6.5
Map3k9	mitogen-activated protein kinase kinase kinase 9	6.4
Gbp5	guanylate binding protein 5	6.3
Pou4f1	POU domain, class 4, transcription factor 1; Brn3	6.2
Ina	internexin neuronal intermediate filament protein, alpha; NF66	6.2

1 p<0.05 identified using SAMMate with EdgeR.

Genes associated with genetic networks identified by IPA are shown in bold.

2 Overlaps CXCL10 and CXCL11 so its upregulation may be due to this overlap.

**Table 4:** Top 15 host genes^1^ repressed in infection.

Gene	Full name	Fold change
Npy6r	neuropeptide Y receptor Y6	−30.6
Rxfp1	relaxin/insulin-like family peptide receptor 1	−30.3
Gm15411^2^	predicted gene 15411	−29.6
**Mc2r**	**melanocortin 2 receptor, adrenocorticotropic hormone receptor**	**−29.3**
Gm867	predicted gene 867	−29.3
4933400A11Rik	RIKEN cDNA 4933400A11 gene	−29.3
AC159008.1 (Musd2)	Mus Musculus type D-like endogenous retrovirus 2	−29.3
A530013C23Rik^2^	RIKEN cDNA A530013C23 gene	−29.1
Cd200r3	CD200 receptor 3	−29.1
Antxrl	anthrax toxin receptor-like	−29.1
8030423F21Rik	RIKEN cDNA 8030423F21 gene	−29.1
**Mup3**	**major urinary protein 1**	**−29.1**
Gm10689	predicted gene 10689	−29.1
4930455H04Rik	RIKEN cDNA 4930455H04 gene	−29.1
4930412B13Rik	RIKEN cDNA 4930412B13 gene	−29.1

1 p<0.05 identified using SAMMate with EdgeR.

Genes associated with genetic networks identified by IPA are shown in bold.

2 lincRNA.

**Table 5:** Top 20 host genes^1^ downregulated in infection.

Gene	Full name	Fold change
**Ggt2**	**gamma-glutamyltransferase 2**	**−5.6**
**Scara5**	**scavenger receptor class A member 5; testis expressed scavenger receptor**	**−5.1**
**Il1r2**	**interleukin 1 receptor, type II**	**−4.7**
E230015J15Rik	RIKEN cDNA E230015J15 gene	−4.5
Gm12963^2^	predicted gene 12963	−4.4
Gpr165	G protein-coupled receptor 165	−4.3
**Clec3b**	**C-type lectin domain family 3, member b**	**−4.3**
Gm15883^2^	Predicted gene 15883	−4.2
Palmd	Palmd	−4.2
**Agtr2**	**angiotensin II receptor, type 2**	**−4.2**
Gm16890^3^	Dsec\GM16890	−4.1
Ahnak2	AHNAK nucleoprotein 2	−4.0
Cyp2f2	cytochrome P450, family 2, subfamily f, polypeptide 2	−3.9
Gm10544^4^	predicted gene 10544	−3.9
Gstm6	glutathione S-transferase, mu 6	−3.8
Gm12575^5^	predicted gene 12575	−3.8
mmu-mir-685.1^6^	microRNA 685	−3.8
Olfr1314	olfactory receptor 1314	−3.7
Snord15a	small nucleolar RNA, C/D box 15A	−3.7
Olfr78	olfactory receptor 78	−3.7

1 p<0.05 identified using SAMMate with EdgeR.

Genes associated with genetic networks identified by IPA are shown in bold.

2 antisense transcripts.

3 recently withdrawn from Mouse Genome Informatics (MGI) database.

4 uncharacterized RNA.

5 lincRNA.

6 microRNA record discontinued.

**Figure 6:**
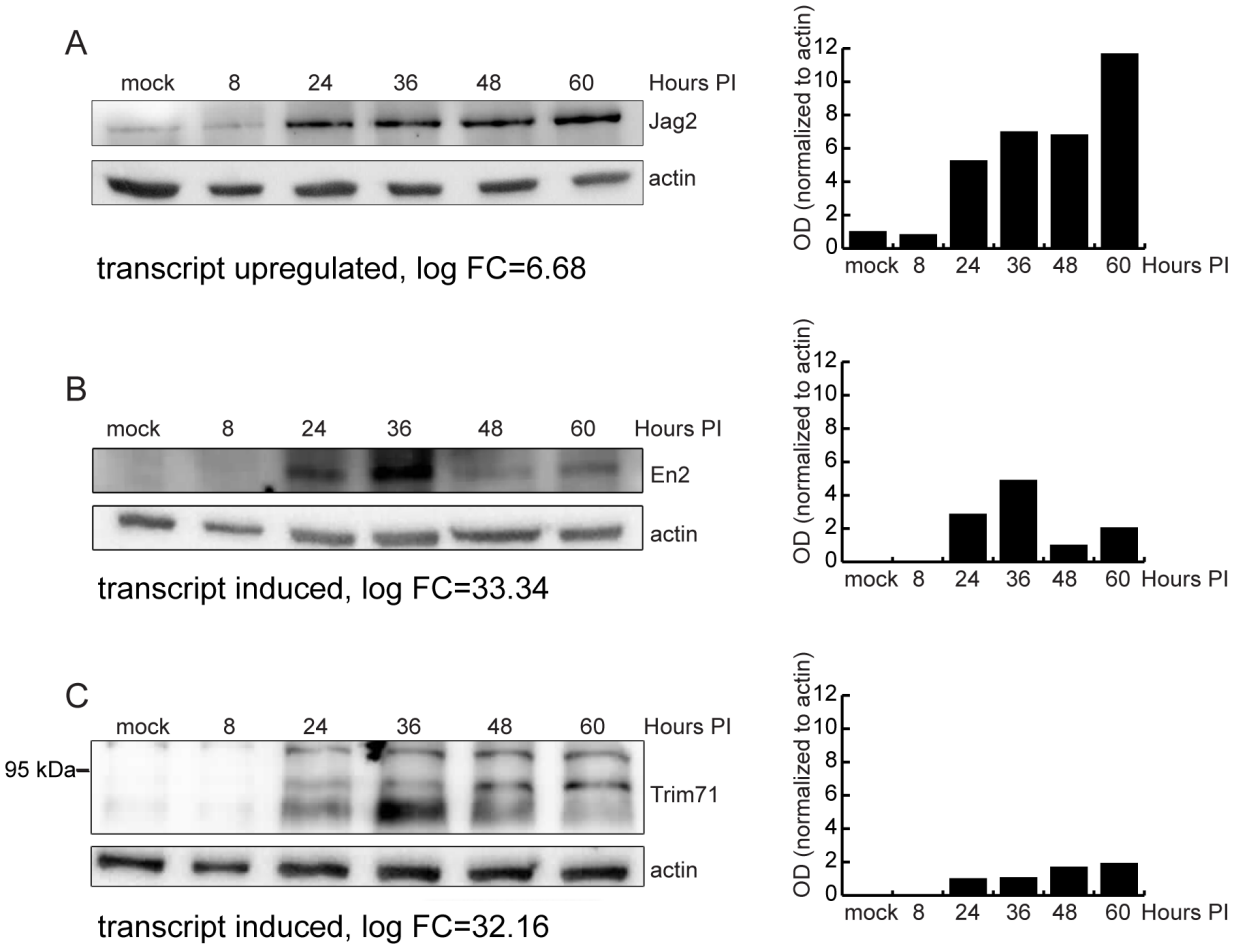
Validation of RNA-Seq analysis of host genes by western blot. (A) Immunoblot analysis of MEF.K (A) or Balb/c MEF (B–C) cell lysates infected with wild-type MCMV. Cell lysates were separated by SDS-PAGE, transferred to PVDF membrane, and probed with antibody to Jag2 (A), EN2 (B) or Trim71 (C). Monoclonal antibody to actin was used as loading control. Bar charts represent relative quantification of proteins. In the case of Trim71 (C where anti-Trim71 antibody detected multiple bands, the bars show quantification of the middle band.

Chemokine ligands dominated the group of the top upregulated genes. Genes encoding proteins with roles in intrinsic cellular defense were also highly upregulated, including *OAS1*, *Mx1*, *Gpb5* and *Rsad2* (a.k.a. *viperin*). There were also a surprising number of genes involved in development, differentiation, and stem cell renewal strongly induced or upregulated by infection, including *FoxA1, Spint1, Lin 28B, En2, Gabrq, Esx1, Trim71, Trp73, Cpne5, Cdh7, Cited 1, Pou4f1*, and *Jag2*. The relevance of these genes, as well as others including *Art3, Ugt8*, and *Trank1* to infection is not clear. We analyzed protein levels of several induced and upregulated transcripts whose relevance to MCMV infection is unknown (**Figure 6**) including the notch ligand Jagged 2, the homeobox-containing transcriptional factor Engrailed 2, and the E3 ubiquitin-protein ligase Trim 71. Protein levels of all proteins tested correlated with their transcript levels in infected BALB/c fibroblasts.

Interestingly, the top repressed and downregulated genes are primarily of unknown relevance to infection, though many are receptor or cell surface molecules (*Npy6R, Rxfp, Mc2r, Cd200r3, Antxrl, Scara5, Il1r2, Agtr2, GPR165*, the olfactory receptor genes, *Olfr1314* and *Olfr78*, and the lectin or lectin-like genes *Clec 3b* and *Reg3A*). MCMV infection also caused repression or downregulation of noncoding (nc)RNAs including the small nucleolar RNA gene, *Snord15A* and genes of unknown function including 3 long intergenic noncoding RNAs (lincRNAs), the miscellanous RNA, *4930412B13Rik*, and 2 antisense transcripts (*Gm12963, Gm15883*). To summarize, while many of the top upregulated genes are associated with host responses to infection, the function of many of the top downregulated and repressed genes during infection are obscure.

### Global gene networks altered by MCMV infection

Genes and their products do not work in isolation but rather form pathways and networks. Even small perturbations in gene expression in a pathway can exert profound influences on eventual processes or functions. Therefore, we analyzed gene lists for shared common pathways. As expected, the top scoring gene networks for all differentially expressed (DE) genes included (i) infectious disease, antimicrobial response, inflammatory response (28 focus molecules); (ii) inflammatory response, cellular development, cell-mediated immune response (27 focus molecules) and (iii) cell morphology, hematological system development and function, inflammatory response (19 focus molecules) (**Table S6A**). These were also top networks when the subset of up- and down-regulated genes were evaluated (**Table S6B**). Also identified were networks associated with cell morphology and hematological system development and function. When this analysis was conducted with only induced and repressed genes, the top networks included cellular development, cell-mediated immune response, cellular function and maintenance, gene expression and embryonic development (**Table S6C**). The relationships among the molecules in top networks for differentially regulated and induced/repressed genes are shown in **Figures Figure S6** and **Figure S7**. Thus, an unexpected outcome of this analysis is that MCMV infection influences a subset of networks controlling development..

**Figure 7:**
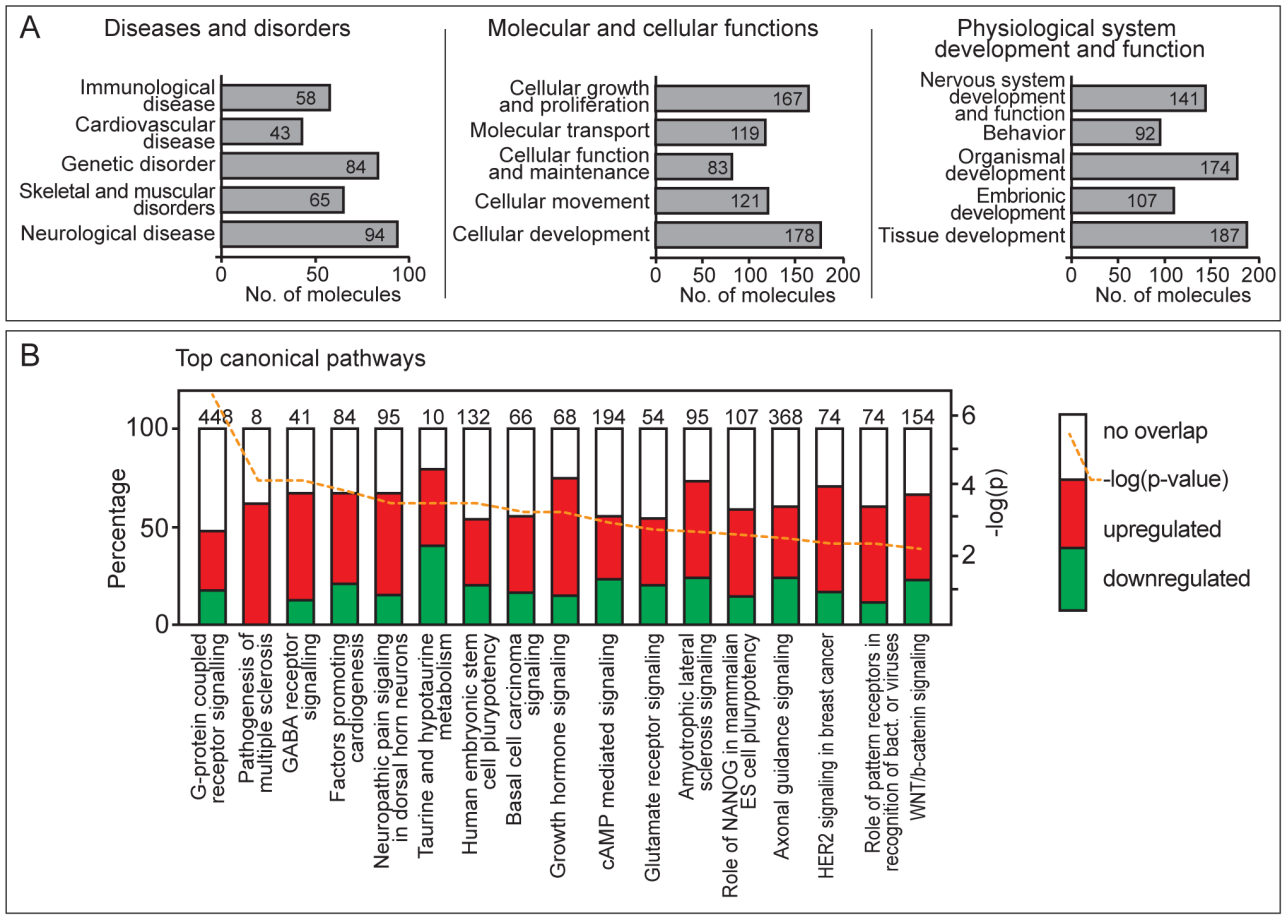
Gene enrichment analysis of differentially regulated mouse genes in MCMV infection. Differentially expressed genes were identified by SAMMate and analyzed with IPA Core Analysis with fold change ratio cutoff of 2. Shown are top diseases and disorders, molecular and cellular functions, and physiological system development and functions (A) and top canonical pathways (B) of DE genes.

The biological functions and/or diseases that were most significant to the molecules in the MCMV-regulated networks are shown in **Figure 7A**. Immunological disease, cardiovascular disease, genetic disorders, and skeletal and muscular disorders ranked as the top bio-functions connected with genes altered by MCMV infection. Among molecular and cellular functions, cell growth and proliferation were the top ranked perturbed functions, consistent with known effects of lytic MCMV infection of cells. Nervous system development and function is at the top of the list of physiological and developmental biofunctions, followed by organismal and tissue development and, surprisingly, behavior with 92 associated genes. DE genes were also evaluated for canonical pathways in the Ingenuity library (**Figure 7B**). The pathways most affected by MCMV included G-protein coupled receptor signaling followed by pathogenesis of multiple sclerosis and GABA receptor signaling. Together, these analyses point to known and expected consequences of infection at the cellular level (i.e., cell growth and proliferation, G-protein coupled receptor signaling) and physiological level (i.e. nervous system development) but also highlight unexpected cell and molecular functions, as well as physiological systems and disorders that may advance the understanding of CMV pathogenesis.

Gene ontology (GO) enrichment using GOrilla ranked lists analysis [31], [32] was also used to analyze DE genes. The full list of enriched GO terms long with associated genes is shown in **Table S7**. GOrilla analysis highlighted processes associated with upregulated genes including cell differentiation, neuron differentiation, regulation of ion transport, and the G-protein coupled receptor signaling pathway. Genes downregulated during MCMV infection were associated with many processes, including regulation of cell shape, adhesion, motility, and the extracellular matrix. Altogether, GOrilla analyses support results of the Ingenuity pathway analysis and suggest novel processes regulated in infected cells, notably suggesting that infection leads to a restructuring of the extracellular environment of the infected cells.

## Discussion

We report a comprehensive analysis of the MCMV transcriptome during lytic infection derived from cloning and sequencing of viral transcripts and next generation sequencing (RNA-Seq). By combining the approaches of RNA-Seq and traditional cDNA cloning as well as northern and RT-PCR analyses in certain complex regions, we were able to construct a comprehensive profile of viral and host transcription during lytic infection. We also investigated the host transcriptome using RNA-Seq combined with differential gene expression analysis, pathway analysis, and gene ontology analysis.

The major findings are as follows: 1) The MCMV transcriptome diverges substantially from that predicted by current annotation; 2) the identification of a novel viral protein specified by the MAT transcript indicates that this transcript functions as an mRNA and a non-coding RNA; 3) the majority of the most abundantly transcribed viral genes are of unknown function; and 4) the host response to infection includes regulation of many host genes and gene networks of unknown relevance to infection.

There are four major findings from the analysis of the MCMV transcriptome. First, we demonstrate novel transcripts of MCMV including novel splice variants, transcripts that map to noncoding regions, and transcripts overlapping multiple genes. Earlier, we reported similar novel transcripts of HCMV through analysis of a classical cDNA library [16]. This study revealed a dramatic increase in the complexity of viral gene products compared to currently available predictions and its findings were later on confirmed by RNA-Seq analysis [17]. A more recent analysis of HCMV translational products [18] by ribosomal footprinting identified over 700 translated ORFs – a strikingly high number compared to annotated genes. This discrepancy is, at least in part, a consequence of the polycistronic nature of HCMV transcripts which appear to code for many more ORFs than previously predicted (internal in frame or out-of-frame ORFs, uORFs) as well as ORFs coming from antisense or dedicated short transcripts. Our analysis demonstrated that the MCMV transcriptome is similarly complex: we identified several regions where multiple 3′ co-terminal transcripts expressed in different temporal phases are being transcribed. Such transcripts have the potential to code for truncated protein forms or even completely new proteins as described for HCMV, suggesting that the size and complexity of the MCMV proteome, like the MCMV transcriptome, is currently underestimated. Accumulation of ncRNAs is also a prominent feature of the cytomegalovirus transcriptomes. Our RNA-Seq analysis shows intense transcription in previously described stable MCMV introns and in intergenic regions, consistent with abundant ncRNAs reported for HCMV and MCMV [16], [17], [33]. These findings have a profound implication for understanding studies of CMV genes functions and underscore the need for transcriptomic maps in addition to genomic maps depicting only ORFs. The functions of many MCMV genes have been elucidated by using deletion mutants [34]. However in a transcriptionally complex region of the genome any deletion will likely impact multiple transcripts and possibly multiple proteins resulting in complex phenotypes.

In line with previous studies [13], we identified novel AS transcripts of MCMV. Interestingly, preliminary estimates in our cloning study indicate that AS transcripts occur at much lower frequency than reported for HCMV [16]. There are likely to be additional AS transcripts of MCMV. Because we did not capture every known sense transcript of MCMV, we may presume that the cDNA cloning study did not capture all AS transcripts. In addition, the RNA-Seq analysis performed in this study was limited by the fact that the methods employed did not provide strand-specific information and could not identify novel AS transcripts. AS transcripts, even those expressed at low levels, may possess noncoding RNA functions and contribute to complexity of the proteome as has described for HCMV [20]. Therefore, further studies are needed to determine the number and nature of AS transcripts derived from MCMV and will be critical to generating definitive transcriptome and proteome maps of this virus. The cDNA library analysis does suggest that the extent of MCMV AS transcription is lower than that described for other herpesviruses, including HCMV. These results are consistent with a strand-specific RNA-Seq experiment performed by Dölken group [15] that also show poor AS transcription in comparison to sense counterparts. Very little antisense transcription was also noted for the anguillid herpesvirus 1 (AngHV1) infecting eels [35], though extensive antisense transcription was reported for other herpesviruses, including KSHV and MHVγ68 [36], [37]. We conclude that different members of the *Herpesviridae* family differ in the extent of antisense transcription during lytic infection.

Second, we observed similar inconsistencies between transcriptomic data and gene annotation for MCMV as previously reported for HCMV [16]. These discrepancies can profoundly impact future studies related to the quantitative analyses of gene expression, interpretation of microarray studies, comparisons to newly sequenced virus strains, and studies using deletion mutant virus strains. The results presented here represent an important first step in re-annotation of the MCMV genome and underscore the utility of transcriptome studies in validating and refining genome annotation for microbial pathogens.

Third, analysis of the MCMV transcriptome revealed the striking abundance of the spliced MAT transcript. This gene is also largely conserved in wild isolates of MCMV (Alec Redwood, personal communication and [38]) and the protein is expressed by wild isolates tested in this study. MAT abundance may reflect its multiple functions. The 3′ untranslated region (UTR) of this transcript facilitates degradation of murine miR-27, establishing that this transcript functions as a noncoding RNA molecule [19], [20]. Members of the alpha, beta, and gamma herpes virus subfamilies all encode for abundant, largely enigmatic noncoding RNAs including the latency associated transcript (LAT) of herpes simplex virus (HSV), EBNA RNAs of Esptein-Barr virus (EBV), the β2.7 transcript of HCMV, the PAN RNA of Kaposi's sarcoma herpes virus (KSHV) and the HSUR transcripts of herpesvirus Samiri (HVS) which also downregulates the cellular miR27 (reviewed in [39]). In addition to the noncoding function of the MAT, we demonstrate that this transcript also encodes for a novel small protein of approximately 17 kDa. To our knowledge, this is the first herpes virus transcript we know of that functions both as a noncoding RNA, and an mRNA that specifies a novel viral protein.

Fourth, a somewhat startling finding from the quantitative RNA-Seq analysis was that after MAT, the most abundant viral transcripts in infected cells are derived from genes without known functions, including *M116*. We report that M116 is a novel spliced transcript predicted to specify a much smaller protein compared to current annotation. These results highlight fundamental gaps in our understanding of basic MCMV biology.

We found that the cDNA library and RNA-Seq approaches yielded remarkably complementary data including identification of novel transcripts and new insights into transcript abundance, despite different biases in each of these methods. For example, while there may be selection bias for isolating transcripts with long tracts of adenosines during cDNA library construction [16], GC content, bias in the sites of fragmentation, primer affinity and transcript-end effects may influence RNA-Seq results [40]. Future RNA-Seq studies may also facilitate novel gene identification as RNA-Seq has now been applied to *ab initio* reconstruction of gene structure [41] using only RNA-Seq data and the genome sequence. However, currently available algorithms are unable to cope with highly dense genomes, such as MCMV and other viral genomes. Until such tools are developed for very dense genomes, RNA-Seq data relies upon comparison to existing gene annotation and other experimental methods for gene structure prediction. In this study, we compared RNA-Seq to currently used annotations but also to the cDNA library study, northern analysis, and RT-PCR studies to identify and validate numerous novel transcripts.

We also report that lytic infection elicits a profound cellular response in fibroblasts. This study identified 10,748 differentially regulated genes. As the number of mouse genes is estimated to be 33,207 [42] we estimate that over 31% of mouse genes are altered in response to infection. Many of the top upregulated and induced genes and gene networks were associated with immune responses to infection, including interferon and interferon-inducible genes such as *phyin1*, a potential activator of p53 [43], the inflammasone regulator *Gpb5* [44] and *Rsad2* (a.k.a. viperin), also known to be induced by HCMV [45].

Inflammatory chemokine ligand genes are also highly upregulated during infection. MCMV encodes virally-derived chemokine homologs specified by the *m131/m129* genes [46], [47] and at least one chemokine receptor homolog, M33 [48]. Numerous host chemokine receptors are also upregulated by infection, suggesting a remarkably complex interplay between MCMV-derived and host derived chemokine signaling during infection. Induction of inflammatory gene networks by MCMV also lends credence to the hypothesis that inflammatory responses link CMV infection to chronic diseases, such as chronic allograft rejection, cardiovascular disease, and cancer [2], [4], [5].

Numerous transcription factors are also induced or upregulated by infection including insulinoma-associated 1 (*Insm1*). Recently, *Insm1* was found to be strongly upregulated by HSV-1 infection and shown to promote HSV gene expression, probably by binding the HSV infected cell protein (ICP)0 promoter. [49]. This raises the intriguing possibility that INSM1 plays a similar role in promoting virus gene expression during MCMV infection. Another induced transcription factor induced at the transcript and protein level is engrailed-2 (EN2). This transcription factor is key to patterning cerebellar foliation during development [50]. We previously described a profound dysregulation of cerebellar development in brains of neonatal mice infected with MCMV [51], suggesting a possible physiological link to regulation of this gene. Another top induced gene was the GABA receptor, *Gabrq*. Glutamate receptor signaling was also identified as significantly impacted canonical pathways in our dataset. In the developing brain GABA and glutamate receptors influence neuronal proliferation, migration, differentiation or survival processes [52]. Whether and how these observations relate to our previous findings that MCMV infection of neonates results in decreased granular neuron proliferation and migration [51] are important areas for future study and may impact our understanding of neurological damage and sequelae associated with HCMV in congenitally-infected infants.

Perhaps most importantly, many top regulated genes, especially downregulated and repressed genes, are associated with functions whose roles in infection are obscure, including many genes of unknown function. Many downregulated or repressed genes are cell surface molecules, host lincRNAs, antisense RNAs, or small nucleolar RNAs. Regulation of lincRNAs was recently observed during infection with severe acute respiratory syndrome coronavirus (SARS-CoV) and influenza virus, and have been suggested to impact host defenses and innate immunity [53]. Further studies to identify the functions of these downregulated and repressed genes and noncoding RNAs during MCMV infection may well provide novel insights into the virus-host molecular interface as well as possible therapeutic targets.

This analysis also revealed immunological disease, cardiovascular disease, genetic disorders and skeletal and muscular disorders as top bio-functions connected with genes altered by MCMV infection. While MCMV involvement in cardiovascular disease is a subject of intensive research, potential involvement in skeletal and muscular disorders is not well documented but may be relevant to the novel observation that MCMV infection of mice with a heterozygous *Trp53* mutation develop rhabdomyosarcomas at high frequency [54].

A primary caveat of RNA-Seq analysis is determining whether changes in gene transcript levels are also reflected at the protein level. This is particularly important as herpesviruses can control protein accumulation at the post-transcriptional, translational, and post-translational levels [55]–[57]. We confirmed that the notch ligand, Jagged 2, is highly upregulated by infection at both the transcript and protein level. Notch signaling is a highly conserved signaling pathway that plays important roles in development, including neurogenesis and differentiation of immune cell subsets [58]. Jagged 2 is also upregulated by the alphaherpesviruses, HSV-1 and Psuedorabies viruses [59]. KSHV and EBV also exploit the notch signaling pathway to facilitate aspects of their life cycle [60] and notch signaling is proposed to influence HSV-2-induced interferon responses [61]. We show for the first time that the betaherpesvirus, MCMV, also influences notch signaling. Dysregulation of Jagged2 as a consequence of MCMV infection is highly interesting since it plays a role in important processes affected by CMV including inner ear development [62], [63], generation of motor neurons [64] and differentiation of immune cell subsets [65], [66].

To summarize, this study has refined the understanding of MCMV gene expression and identified new areas of research to advance our understanding of the host response to these ancient viruses. We describe what is to our knowledge, the first herpes virus transcript that functions as both a noncoding RNA that limits accumulation of cellular miRNAs, and an mRNA that specifies a protein. This study also revealed novel features of the host response to infection. Perhaps most importantly, this study identified many virus and host genes of unknown function that are regulated during infection. It is highly likely that further study of these genes may lead to breakthroughs in the understanding and treatment of cytomegalovirus-related diseases.

## Materials and Methods

### Cells, viruses and infection conditions

Primary mouse embryonic fibroblasts (MEFs) from BALB/c or Balb.K mice were prepared and maintained as described [67] and used between passages 3–8. Immortalized murine BALB.K MEFs, (MEF.K) [68] and SVEC4-10 (ATCC CRL-2181) were used for immunoblot studies. MCMV Smith strain (ATCC VR-1399) was propagated and titrated on primary Balb/c MEF by standard plaque assay as described in detail in [69]. Wild type MCMV isolates K181 (GenBank acc no: AM886412.1), C4D, K6 and WP15B (GenBank acc no: EU579860.1) [38] were a kind gift from A. Redwood (University of Western Australia, Australia). Construction of the Δ7S3, Δm167, Δm168, Δm169, Δm170, and m169-mut mutant viruses were previously described and were generated by ET-cloning [70] using the full-length MCMV BAC pSM3fr [71]. The double deletion mutants (Δm168Δm169 and Δm169Δm170) were constructed exactly as described previously [20]. Primers for construction of the double deletion mutants are also as described [20] using the forward primer for the first gene and reverse primer for the second gene. All infections were conducted by exposing cells to 0.3 PFU/cell followed by centrifugal enhancement for 30 minutes at 800 g, as described in [69].

### Extraction of MCMV genomic DNA

Smith MCMV infected cBalb/c MEFs were harvested 72 h post infection and viral DNA was isolated as described [16].

### Construction of MCMV cDNA libraries

RNA was extracted from Smith MCMV infected Balb/c MEF at 4, 8 and 12 hrs after infection (IE library); 16, 24 and 32 hrs after infection (E library); and 40, 60 and 80 hrs after infection (L library). No drug was used to select for different temporal classes of transcripts and equal amounts of RNA from each time point were pooled prior to library construction. cDNA libraries were generated as described previously for HCMV [16] by following the instruction manual for the SuperScript Plasmid System with Gateway Technology for cDNA Synthesis and Cloning (Invitrogen) with some minor modifications. Briefly, total RNA was isolated using the TRIZOL Reagent (Invitrogen, CA, USA). A poly(T)-tailed PacI primer-adapter was used for first-strand cDNA synthesis (5′-GCGGCCGCTTAATTAACC(T)^15^-3′). After second-strand synthesis, an EcoRI-PmeI adapter was added to the 5′ end and cDNAs were cleaved with EcoRI and PacI. The EcoRI-PmeI adapter was generated by annealing following oligonucleotides: 5′-AATTCCCGCGGGTTTAAACG-3′ and 5′-Pho-CGTTTAAACCCGCGGG-3′. cDNA fragments were inserted into a modified pcDNA3.1(+) previously digested with EcoRI and PacI and transformed into XL1-Blue Supercompetent *E. coli* cells (Stratagene, CA, USA).

### Screening of cDNA library and sequencing

Positive selection of viral cDNA clones was performed as described previously [16]. *Mse* I-digested genomic MCMV DNA was labeled using a DIG High Prime DNA Labeling Detection Starter Kit II (Roche Applied Science) and used to identify virally-derived cDNA clones. Plasmids harboring cDNA clones that reacted with probe were isolated and sequenced from the 5′ end using T7 primer for pcDNA3.1(+) or the 3′ ends using primer (5′GCACCTTCCAGGGTCAAGGAAG) or standard poly (T) primers at the OSU Plant-Microbe Genomics Facility. Sequences were compared to the MCMV Smith strain genome [GenBank accession no. NC_004065] using mega BLAST.

### Next generation sequencing – Library preparation, alignment and analysis

Total RNA was extracted from Balb/c MEF cells cultured in 100 mm^2^ petri dishes and exposed to 0.3 PFU/cell of the MW 97.01 strain of murine cytomegalovirus or mock-infected. At 4, 8, 12, 16, 24, 32, 40, 60 and 80 hours after infection, RNA was isolated using TRIZOL Reagent. RNA integrity was assessed on Agilent Bioanalyzer and only samples with RNA index values of at least 9 were used. Equal amounts of RNA from each time point were pooled (0.3 µg of RNA per time point) and treated with DNaseI. Libraries were prepared with Illumina TruSeq RNA kit according to manufacturer's instructions and sequenced on Illumina Genome Analyzer IIx as single-end 36 bp reads. The Illumina TruSeq RNA kit employed does not allow for strand-specific information to be derived from the sequence data. Datasets are available at the National Center for Biotechnology Information (NCBI) Sequence Read Archive (SRA) accession no. SRR953479 (sequence reads from MCMV-infected MEFs) and accession no. SRR953859 (sequence reads from mock-infected MEFs).

Reads were aligned to mouse (NCBI37/mm9 assembly) and MCMV genome (GenBank acc.no. NC_004065.1) using ELAND aligner or Bowtie aligner (for comparison with data provided by Lars Dölken). It is important to note that both ELAND and Bowtie aligners do not map splice junctions and thus give concordant results. Alignments were visualized using Integrative Genomics Viewer (http://www.broadinstitute.org/igv/) [72]. Differential gene expression was assessed by calculating RPKM (reads per kilobase of million mapped reads (RPKM) using SAMMate 2.6.1. release with EdgeR (http://sammate.sourceforge.net/) [73]. Gene ontology (GO) enrichment analysis was performed on filtered lists of differentially expressed genes (p<0.05) using GOrilla ranked lists analysis [31], [32]. Ingenuity Core Analysis (Ingenuity Systems, www.ingenuity.com) was used for gene interaction network and canonical pathway analysis. Gene lists were filtered for statistically significant differentially expressed genes (p<0.05) and a fold change cutoff of 2 was set to identify molecules whose expression was significantly differentially regulated. For network generation, these molecules (Network Eligible molecules), were overlaid onto a global molecular network developed from information in the Ingenuity Knowledge Base based on their connectivity. The Functional Analysis of a network identified the biological functions and/or diseases that were most significant to the molecules in the network. Right-tailed Fisher's exact test was used to confirm that biological functions and/or disease assigned to data sets were not due to chance. The nature of individual DE genes was also investigated using the Mouse Genome Informatics databases (http://www.informatics.jax.org/) [74] and Entrez Gene (http://www.ncbi.nlm.nih.gov/gene) [75].

### Northern blot analysis

RNA was isolated using Trizol reagent from mock or MCMV-infected Balb/c MEF at 24 hours (MAT) or 10, 30 and 60 hrs after infection. RNA (1 µg/lane or 10 µg/lane (MAT)) was separated by formaldehyde agarose gel electrophoresis and transferred to positively charged nylon membrane and crosslinked by UV irradiation. Membranes were reacted to DIG-labeled probes overnight at 67°C. For MAT detection, a DIG labeled double-stranded DNA probe was made using fragments corresponding to the MAT gene sequences derived from cDNA library clones E1, E125 and E134 using Roche's DIG-High Prime DNA Labeling and Detection Starter Kit I. For all other northern blots, single-stranded DIG-labeled RNA probes were used generated using Roche's DIG Northern Starter Kit. Antisense probes were generated by in vitro transcription from T7 promoter present in pcDNA3.1 plasmids containing cDNA clones that harbor the desired gene fragments (Table 1). Therefore antisense probes are identical to transcripts cloned in cDNA library and can detect transcripts antisense to cDNA clones. To generate sense probes, T3 promoter was added to 5′ end of complimentary strand of the gene fragments used for antisense probes by PCR (Table 1). The PCR fragments were then in vitro transcribed and DIG labeled using T3 RNA polymerase. Care was taken to generate sense probes of length comparable to corresponding antisense probes.

### Generation of the antibody against m169

The m169 gene sequence was amplified by PCR using viral DNA isolated from MCMV BAC pSM3fr using following primers: F: 5′-TTTTTGGATCCATGAGCAACGCGGTCCCGTTC-3′ and R: 5′- TTTTTCTGCAGTCATCACGGGGGGCACCTACC-3′, reacted with BamHI and HindIII (New England Biolabs), inserted into pQE30 expression vector and introduced to *E.coli* Bl21 pREP4 strain (Qiagen). The protein was induced according to manufacturers' instructions and purified on a His-tag column. Purified protein was used for immunization of Balb/c mice and antibody titer in blood serum was measured by ELISA. When antibody titer in serum reached adequate levels, animals were sacrificed, their spleens isolated and fused with SP2/O cells. Supernatants from motherwells were tested by immunoblot blot on purified MAT protein and positive wells were rescreened by immunoblot using lysates from MEFs infected with WT, Δ7S3, Δ168-169, Δ169-170, Δm168, Δm169 and Δm170 mutants as described below.

### Validation of splicing by PCR

RNA from Mock- or MCMV-infected cells isolated for northern blots was also reverse transcribed using oligo-dT primers (ProtoScript M-MuLV *Taq* RT-PCR Kit, New England BioLabs). No reverse transcriptase (-RT) controls were run in parallel. Splicing was then verified by PCR amplification using primers that flank putative introns (M116; F: CTTCATCGGATTCGGAGGC; R: TGTTGTTGTCGACGTCTGATGTG; m71–m75; F: ATCTCCTCTGCCTCCGACCTC, R: CGATGTCATCTTGGAATCCGACGA; m72–m75; F: CCGGATACGACCGTCAGC, R: CGATGTCATCTTGGAATCCGACGA) using Phusion high fidelity polymerase (New England BioLabs).

### Immunoblot analysis

Mock-infected or MCMV-infected primary MEFs, or murine cell lines (MEF.K, SVEC4-10) were lysed in RIPA buffer. Protein lysates were separated by SDS-PAGE and transferred to PVDF. MAT protein was detected with anti-m169 antibody described above, Jag2 with antibody N-19 (Santa Cruz), Engrailed 2 with En2 PA5-14363 antibody (Thermo Scientific), Trim71 with PA5-19282 (Thermo Scientific), and actin with antibody C4 (Millipore) followed by peroxidase-labeled secondary antibodies (Jackson ImmunoResearch or Abcam). Proteins were visualized using Amersham ECL Prime Western blotting reagent (GE Healthcare) and quantified using ImageJ software (http://rsbweb.nih.gov/ij/).

## Supporting Information

Dataset S1 **Comparison of sensitivity and temporal gene expression data from this study to previous microarray studies of MCMV (S1A and S1B) and Comparison of RPKM values in Marcinowski** ***et al.*** **(2012) and this RNASeq experiment (S1C).**
(PDF)

Dataset S2 **Spliced cDNA clone overlapping M116 and comparison of predicted protein to current annotation.**
(PDF)

Figure S1 **RNA-Seq profiles comparison.** RNA-Seq data from total RNA obtained from MCMV infected NIH-3T3 fibroblasts from 25 and 48 hrs PI sequenced by Dölken group (GSE35833) was aligned against MCMV genome (gB acc no NC_004065.1) using Bowtie aligner and visualized in IGV in comparison to our RNA-Seq data. The view of the complete genome is shown at the top with 4 areas magnified below (labeled A–D) and the number of reads displayed are noted on the side. Since viral genes display a wide range of expression levels, the whole genome view is shown in wide data range (upper panel) more suitable for displaying highly transcribed regions and a narrowed data range (lower panel) that is more suitable for less transcribed regions. As can be seen, the profiles of the compared alignments are remarkably similar, the only differences being abundance of certain transcripts which are due to different time points analyzed in comparison to the pooled data of our RNA-Seq and significantly greater depth of at least one order of magnitude of our data in comparison to Marcinowski data.
(TIF)

Figure S2 **Analysis of the** ***m20–19*** **region.** Balb/c MEF cells were infected with BAC derived Smith virus and harvested 10, 30 and 60 hrs post infection. Total RNA was separated by denaturing gel electrophoresis, transferred to nylon membrane and incubated with probe generated by in vitro transcription from T7 promoter of L57 [A; probe should detect predicted m19(S) transcripts] or probe generated by in vitro transcription from T3 promoter of IE205 transcript [probe should detect m20(S)-m19(AS) transcripts]. RNA integrity and loading was evaluated by inspecting 28S (not shown) and 18S rRNA bands under UV light after transfer to membrane. Predicted genes (Rawlinson's annotation) are depicted as empty arrows, while thin black arrows show longest transcripts cloned in our cDNA library as well as clones used to generate probes (marked with *). 3′ ends of transcripts are marked with arrowheads. The nucleotide coordinates relative to Smith sequence (NC_004065.1) of isolated transcripts are given below thin arrows, while the names of the clones are written above. Gray histograms showRNA-Seqreads aligned to MCMV genome. Maximal possible exposure times were used to ensure even low abundance transcripts are detected and are noted on the blots.
(TIF)

Figure S3 **Verification of M116 splicing by PCR.** Schematic of the M116 gene region and clone L29 (A) and PCR analysis of the splice site (B). In (A) Predicted genes (Rawlinson's annotation) are depicted as empty arrows, while the thin black arrow depicts clone L29 with red arrows depicting the primers used in (B). The 3′ ends of transcripts are marked with arrowheads. The nucleotide coordinates relative to Smith sequence (NC_004065.1). Gray histograms showRNA-Seq reads aligned to MCMV genome. RNA isolated from WT infected Balb/c MEF used in northern blots was reverse transcribed with oligo-dT primers, and then amplified with primers specific for M116 that flank the putative intron (marked by red arrows). No RT controls were run in parallel. Spliced cDNA clones and viral DNA were used as spliced and unspliced amplification controls, respectively.
(TIF)

Figure S4 **Analysis of the** ***m71-m75*** **region spliced transcripts by northern blot and PCR.** Balb/c MEF cells were infected with BAC derived Smith virus and harvested at indicated times post infection (A and B). Total RNA was separated on denaturing gel electrophoresis, transferred to nylon membrane and incubated with probes specific for S and AS transcripts. RNA integrity and loading was evaluated by inspecting 28S (not shown) and 18S rRNA bands under UV light after transfer to membrane. Predicted genes (Rawlinson's annotation) are depicted as empty arrows, while thin black arrows show longest transcripts cloned in our cDNA library as well as clones used to generate probes (marked with *). Images of northern blots are shown using probes derived from the *M72* region in (A) and the *m74* in (B). The 3′ ends of transcripts are marked with arrowheads. The nucleotide coordinates relative to Smith sequence (NC_004065.1) of isolated transcripts are given below thin arrows, while the names of the clones are written above. Thin gray lines show isolated transcripts that cannot be detected with the probe. Gray histograms showRNA-Seqreads aligned to MCMV genome. Maximal possible exposure times were used to ensure even low abundance transcripts are detected and are noted on the blots (A and B). In (C), the m71–75 spliced transcript and one of two possible m72–75 spliced transcripts were verified by PCR amplification using primers that flank their putative introns. RNA isolated from WT infected Balb/c MEF used in northern blots was reverse transcribed with oligo-dT primers, and then amplified with primers specific to m71–m75 or m72–m75 spliced transcripts (marked by red arrows). No RT controls were run in parallel. Spliced cDNA clones and viral DNA were used as spliced and unspliced amplification controls, respectively. In (D), the position of exon 1 of *m60* Reported by Scalzo et. al. [23], is compared to RNA-Seq data for this genomic region.
(TIF)

Figure S5 **Northern blot analysis of the** ***M100-M103*** **region.** Balb/c MEF cells were infected with BAC derived Smith virus and harvested at indicated times post infection. Total RNA was separated on denaturing gel electrophoresis, transferred to nylon membrane and incubated with probes specific for S and AS transcripts overlapping *M102* (A), *M100* (B) and *M103* (C) genes. RNA integrity and loading was evaluated by inspecting 28S (not shown) and 18S rRNA bands under UV light after transfer to membrane. Predicted genes (Rawlinson's annotation) are depicted as empty arrows, while thin black arrows show longest transcripts cloned in our cDNA library as well as clones used to generate probes (marked with *). 3′ ends of transcripts are marked with arrowheads. The nucleotide coordinates relative to Smith sequence (NC_004065.1) of isolated transcripts are given below thin arrows, while the names of the clones are written above. Thin gray lines show isolated transcripts that cannot be detected with the probe. Gray histograms showRNA-Seqreads aligned to MCMV genome. Maximal possible exposure times were used to ensure even low abundance transcripts are detected and are noted on the blots.
(TIF)

Figure S6 **Graphical representation of top 3 genetic networks for differentially regulated genes.** Upregulated genes are shown in red, while downregulated are shown in green. Level of differential expression is represented by color saturation with most dramatically changed genes being shown in the most saturated color (strong red or green). These overlapping genetic networks are associated with (i) cell-mediated immune response, cellular development, cellular function and maintenance (30 focus molecules); (ii) infectious disease, antimicrobial response, inflammatory response (27 focus molecules) and (iii) antimicrobial response, inflammatory response, gene expression (24 focus molecules) (see Supplemental table S5).
(TIF)

Figure S7 **Graphical representation of top 5 genetic networks for genes induced or repressed by infection.** Induced genes are shown in red, while repressed are shown in green. Level of differential expression is represented by color saturation with most dramatically changed genes being shown in the most saturated color (strong red or green). These overlapping genetic networks are associated with various developmental processes (see Supplemental table S6).
(TIF)

Table S1 **MCMV transcripts identified in this study compared to current NCBI Reference Sequence Gene Annotation.**
(PDF)

Table S2 **cDNA clones isolated in this study and their characteristics.**
(XLS)

Table S3 **Comparison of experimental and in silico data used for viral gene identification and RPKM values of currently annotated genes.**
(XLS)

Table S4 **Spliced transcripts of MCMV.**
(PDF)

Table S5 **Differentially expressed mouse genes with p<0.05 determined by SAMMate with EdgeR.**
(XLS)

Table S6 **Gene networks and associated genes identified by IPA.**
(XLS)

Table S7 **GOrilla ranked list analysis of DE mouse genes.**
(XLS)
