# Supplementary material for: Dual Analysis of the Murine Cytomegalovirus and Host Cell Transcriptomes Reveal New Aspects of the Virus-Host Cell Interface
Source: PLoS Pathog. 2013 Sep 26;9(9):e1003611. doi: 10.1371/journal.ppat.1003611 (PMC3784481; doi:10.1371/journal.ppat.1003611)
Supplement: Dataset S1 — Comparison of sensitivity and temporal gene expression data from this study to previous microarray studies of MCMV (S1A and S1B) and Comparison of RPKM values in Marcinowski et al. (2012) and this RNASeq experiment (S1C). (PDF) [file ppat.1003611.s001.pdf]

Dataset S1.

S1A. Temporal Analysis of cDNA Clones Isolated in this Study Compared to Previous Microarray Analysis.

S1B. RPKM Values of Predicted Genes not Detected by Previous Microarray Studies.

S1C. Comparison of RPKM values in Marcinowski *et al.* (2012) and this RNASeq experiment.

References

Lacaze, P., T. Forster, et al. (2011). Temporal profiling of the coding and noncoding murine cytomegalovirus transcriptomes. J Virol **85**(12): 6065-6076.

Tang, Q., E. A. Murphy, et al. (2006). Experimental confirmation of global murine cytomegalovirus open reading frames by transcriptional detection and partial characterization of newly described gene products. J Virol **80**(14): 6873-6882.

Marcinowski L, Lidschreiber M, Windhager L, Rieder M, Bosse JB, et al. (2012) Real-time transcriptional profiling of cellular and viral gene expression during lytic cytomegalovirus infection. PLoS Pathog **8**: e1002908.

Dataset S1A. Temporal Analysis of cDNA Clones Isolated in this Study Compared to Previous Microarray Analysis

Viral cDNA clones in this study were derived from 3 libraries, IE, E and L, representing pooled time points from each of these temporal classes. Generally, temporal assignment of cDNA clones in this study agrees with that reported by Lacaze and colleagues (Lacaze et al., 2011). A comparison of temporal assignment of cDNA clones to that reported by Lacaze and colleagues is presented in the Table A, below. Discrepancies were observed including temporal assignments of m20(S), m19(AS), M49, M72(AS), M73, M78, M97, m119.3, M113-M114, m133 Ex1, m132 Ex2, and m131. Some discrepancies may reflect the fact that more time points were represented in libraries of this study. The M116 gene was not detected by Lacaze and colleagues, but was abundant in our study, with a total of 15 clones isolated from early and late gene libraries.

Also consistent with the Lacaze study, the cDNA library analysis suggests very low levels of ie1 or ie3 expression. We detected only 2 clones of ie1 (m123Ex2) in L library, no 128ex3 (ie2) clones, and just one 122ex5(ie3) in L library. By RNA-Seq analysis, an RPKM value of 5070 was found gp114¸(ie1/ie3 exon 2) and 1626 for gp120 (m128Ex3; ie2). For comparison, an RPKM of 4968 was found for gp143 (m152), which was not isolated in cDNA library. For perspective, the average RPKM for all genes using annotation derived from the NCBI reference sequence (NC_004065) is 8015 (standard deviation of 38,536) [If MAT is excluded from this calculation, the average RPKM for all genes using annotation derived from the NCBI reference sequence (NC_004065) is 4275 (standard deviation of 8666)]. Therefore, relative to other viral genes, both the cDNA library analysis and RNA-Seq analysis show average to below average levels of transcription for ie1 and ie3 gene expression, consistent with studies by Lacaze and colleagues.

S1A. Temporal Analysis of cDNA Clones Isolated in this Study Compared to Previous Microarray Analysis

Gene ID NC_004065.1 (MuHV1)	Overlapping Genes (orientation[1])	Strand	No. of Clones	Libraries	Temporal comparison (hrs P.I.)[2]
gp004	m04 (S)	+	7	4IE,E,L	6.5
gp006	m06 (S)	+	6	4IE,E,L	6.5

gp008	m08 (S)	+	1	L	6.5
gp015, gp016	m15, m16 (S) spliced	+	1	E	6.5
	m15, m16 (S)	+	4	2L, IE, E	6.5
gp017	m17 (S)	-	2	IE	6.5
gp018	m18 (AS) spliced	+	1	L	6.5
gp018	m18 (AS)	+	1	L	6.5
gp019	m19 (AS)	-	1	L	
gp020, gp019	m20 (S), m19(AS)	-	3	IE,E,L	24
gp026	M25(S)	+	6	4L,2E	24
gp027, gp028	m25.1 (S)	-	3	2E,1L	6.5
gp027, gp028	m25.1, m25.2 (S)	-	1	E	6.5
gp34	m29 (S), m29.1 (AS)	-	1	E	24
gp036	m30, M31 (S)	+	1	E	6.5
gp038	M32(S)	-	6	3E, 3L	24
gp039	M34 (S) spliced	+	1	E	6.5
gp040	M35 (S)	+	2	E	24
gp040, IGR[3]gp040-gp041	M36, M36 Ex2 (S/AS)	-	1	L	6.5
gp041	M37 (S)	-	2	1IE, 1L	6.5
gp045	m41 (S)	-	2	L	6.5
gp045, gp046	m42, m41 (S)	-	4	3L, IE	6.5
gp045, gp046	m42, m41 (S) spliced	-	1	IE	6.5
IGR[5]gp046-047, gp047	m42 (S)	-	1	1L	6.5
gp047	M43 (S)	-	9	6E, 2L, 1IE	6.5
gp047, gp048	M44, M43 (S) spliced	-	1	IE	6.5
IGR gp048-gp049	M45, M44 (S)	-	1	L	6.5
IGR gp048-gp049, gp049	M45 (S)	-	1	E	6.5
gp051	M47(AS), M46(S)	-	1	L	

gp053	m48.2 (S), m48.1 (AS)	-	3	2L,1E	6.5
gp053, gp054	M49 (S), m48.2 (S), m48.1 (AS)	-	18	10L, 8E	
gp054	M49 (S)	-	2	1E, 1L	24
gp054	M50, M49 (S)	-	1	L	24
gp058	M53 (S)	+	4	3L, 1E	24
gp058 AS	M53 (AS)	-	1	IE	ND
IGR gp058-gp059	M54 (S)	-	2	1IE, 1L	6.5
go059, IGR gp058-gp059	M55, M54 (S)	-	2	L	24
gp060	M69 (S)	-	1	E	24
gp066, IGR AS gp069-gp070	M71, IGR m74-M75 (S)	+	1	E	24
gp067	M72(AS), M73(S)	+	2	1E, 1L	24
gp067, IGR gp069-gp070	M72 (S), M73(AS), IGR m74-M75	+	4	L	
gp068, IGR gp069-gp070	M72 (S), M73(AS), IGR m74-M75 (alternate splice)	+	1	L	
gp068	M72 (AS)	+	1	L	24
gp069	M74 (AS)	+	1	L	48
IGR gp069-gp070	IGR m74-M75	+	1	L	
gp071,gp073	M76(AS), M78(S)	+	1	E	
gp073	M78 (S)	+	8	7IE, 1L	24
gp073	M78 spliced (S)	+	1	E	24
IGR gp073-gp074	M79 (AS)	+	1	L	
gp075	M80 (S)	+	8	4E, 2L, 2IE	6.5
gp075	M80 spliced (S)	+	1	L	6.5
gp076	M82 (S)	-	4	3E, 1L	6.5

IGR gp078-gp079, gp079	IGR M84-85, M85 (S)	-	2	L	24
gp081	M88 (S)	+	1	L	24
gp084, gp085	M92 , M93, M94 (S)	+	1	L	
gp085	M93 (S)	+	1	L	24
gp085, gp086 spliced 1	M93, M94 spliced1 (S)	+	1	L	24
gp085, gp086 spliced 2	M93, M94 spliced2 (S)	+	1	L	24
gp085, gp086	M93, M94 (S)	+	4	2L, IE, E	6.5
gp086	M94 (S)	+	3	2L, E	6.5
gp088, gp089	M95, M96 (S)	+	1	L	24
gp089	M96 (S)	+	1	E	24
IGR gp089-gp099, gp099	M97 (S)	+	1	IE	24
gp092	M98, M99 (S)	+	8	5L, 3E	6.5
gp093	M100 (S)	-	6	3E, 3L	24
gp094	M102 spliced (S)	+	1	IE	6.5
gp094	M102 (S)	+	3	2E, IE	6.5
gp095	M103	-	2	L	24
gp097	M105	+	2	L	6.5
gp098	m106 (S)	-	1	E	24
gp098, IGR gp098-gp099	IGR m106-m107, m106 (S)	-	1	L	24
gp098, IGR gp098-gp099	IGR m106-m107, m106 spliced (S)	-	3	3E, 1L	
IGR gp098-gp099	IGR m106-107	-	1	L	
IGR gp098-gp099, gp099	m108 (S)- m107 (AS)	-	1	E	24
gpM112, gpM113	M112, M113 spliced (S)	+	1	E	24

gpM112, gpM113	M112 Ex1, M113, M112 Ex2, M112 Ex3 (last exon in IGR M112 Ex3-M114) (S)	+	1	L	
gpM113	IGR-m112Ex3-M114 (S)	+	1	E	
gpM113	M113 (S)	+	1	L	48
gpM113, IGR gpM113, gp101	M113, M114 (S)	+	1	IE	24,48
gp101	M114 (S)	-	1	L	24
gp101, gp102	M114, M115 (S)	-	1	L	24
gp103	M116 spliced (S)	-	8	5E, 3L	
gp103	M116 (S)	-	15	9L, 6E	nd
gp106	m119, M118 (S)	-	1	E	6.5
gp107, gp108	m119.1, (S)	-	1	L	24
gp107, gp108, gp109, IGR gp109-gp110	m119.3, m119.2, m119.1(S)	-	7	4E, 2L, 1IE	6.5
gp108, gp109 (AS)	m119.2, (S) m119.3 (AS)	+	1	IE	6.5
gp107, gp108, IGR gp108-gp109	m119.3, m119.2 (S)	-	10	6L, 4E	6.5
gp109	m119.3 (S)	-	2	E	48
IGR gp108-gp109	IGR m119.3 - m119.4 (S)	-	1	E	24
gp108, gp109, gp110, gp111 (AS), gp112	m120(S), m119.5(AS), m119.4 (S),m119.3(S), m119.2 (S)	-	2	IE, E	24
gpM122Ex5	M122 Ex5 (S)	-	1	L	24
gpm123Ex4	m123 Ex4 (S)	-	2	L	6.5

gpM122Ex5, gpm123Ex4, gpm123Ex3, gp114, gp114ex2, gp115	IGR (m124.1 and m125), m123Ex2, m123 Ex3,m122 Ex5 (S)	-	1	E	24
gp121, gp122	m131 (S) - m129 (AS)		1	E	
gpm132Ex2, gp124	m133 Ex1, m132 Ex2, m131 (S)	-	3	2IE, 1L	24
gp123, gpm132Ex2	m132 Ex2 - m131 (S)	-	2	E, L	24
gp124	m133 Ex1 (S)	-	1	IE	
gp128	m137 (AS)	+	1	IE	6.5
IGR gp128-gp129, gp129	m138, m137 (S)	-	1	L	6.5
gp129	m138 (S)	-	12	6E, 3IE, 3L	
gp130	m139 (S)	-	1	IE	24
gp133	m142 (S)	-	2	IE, L	
IGR gp135, gp136	m145 (S)	-	6	3E, 3L	24
gp140	m149 (AS), m150 (S)	-	1	L	ND
gp141, gp142	m150, m151 (AS)	+	1	IE	ND
gp142	m151 (S)	-	1	E	ND
gp145	m154 (S)	-	1	IE	6.5
gp146	m155 (S)	-	3	2IE, L	24
gp147	m156, m155 (S)	-	2	E	24
IGR gp149-gp150, gp150	m159 A (S)	-	1	L	24
gp150	m159 B (S)	-	1	L	
gp151	m160 (S)	-	1	L	6.5
gp151	m160, m161 (S)	-	1	L	24
gp154	m163 (S)	-	1	L	6.5
gp154	m164 - m162 (S)	-	1	IE	6.5
gp154, gp155	m164, m163 (S)	-	14	10L, 3E, 1IE	6.4
gp157	m166 (S)	-	5	3IE, E, L	6.5

gp157, gp158	m167, m166 (S)	-	1	L	6.5
gp158, gp159, gp160	IGR m167-m168, m168 (AS), m169 (S), IGR m169-m170		138	28IE, 57E, 53L	6.5

[1] Sense (S) or antisense (AS) relative to annotated gene.
[2] Comparison to earliest time post-infection transcript was detected by Lacaze et al., 2011.
[3] IGR, Intergenic region
ND, not detected

S1B. RPKM Values of Predicted Genes not Detected by Previous Microarray Studies.

A microarray study conducted by Tang and colleagues (Tang et al., 2006) identified novel ORFs and several were also detected in the cDNA library analysis including m166.5 (1 clone), m132.1 (5 clones) and m84.2 (2 clones).

Studies by both Lacaze and colleagues and Tang and colleagues failed to detect transcripts from numerous annotated genes. Lacaze and colleagues did not detected M44, M70, M75, m135, m143, m144, m153 and m157. We also failed to isolate transcripts from these ORFs in our cDNA library with the exception of M44. Genes whose expression were not detected in the microarray analysis conducted by Tang and Maul, include m01, m19, m26, m22, m69.1, m70, m117.1, m119.5, m126, m127, m129, m134, m144, m150, m165, m170. Of these, we did detect one large clone overlapping m129-131 and one clone overlapping m150 (m150, m151(AS)) in the cDNA library.

Tang and Maul reported the following ORFs as negative by both PCR and microarray analysis: m21, m44.1, m58, m107, m124.1, m125, m130, m141.1, m148, m149, m151, m157, m165.1. The cDNA library in this study did include m107 (4 clones) and m151, however, the clone overlapping m151 was in the antisense orientation to the predicted ORF.

In contrast, most genes not detected by microarray analyses or not represented in this cDNA library were nevertheless detected by RNA-Seq analysis. The RPKM values for each of the undetected predicted genes in the Tang and Lacaze studies are presented in Table B, below. The possible exceptions include m01, m150 and M170, all of which have RPKM values below 200. We conclude that RNA-Seq provides a more sensitive level of detection for analyzing viral gene expression.

S1B. RPKM Values of Predicted Genes not Detected by Previous Microarray Studies.

ORF	ORF	RPKM in MEF	Study
m01	gp001	106.80	Tang et al.
m19	gp019	1349.16	Tang et al.
m22	gp022	457.56	Tang et al.
M26	gp031	883.12	Tang et al.
M44	gp048	6306.94	Lacaze et al.
m69.1	gp064	895.00	Tang et al.
M70	gp065	505.11	Tang et al., Lacaze et al.
M75	gp070	890.93	Lacaze et al.
m117.1	gp105	443.78	Tang et al.
m119.5	gp111	4333.93	Tang et al.
m126	gp118	969.84	Tang et al.
m127	gp119	687.67	Tang et al.
m129	gp121	735.97	Tang et al.
m134	gp125	793.00	Tang et al.
m135	gp126	698.47	Lacaze et al.
m143	gp134	883.56	Lacaze et al.
m144	gp135	322.06	Tang et al., Lacaze et al.
m150	gp141	171.54	Tang et al.
m153	gp144	833.98	Lacaze et al.
m157	gp148	2832.82	Lacaze et al.
m165	gp156	561.84	Tang et al.
m170	gp161	127.10	Tang et al.

Table S1C. Comparison of RPKM values in Marcinowski *et al.* (2012) and this RNASeq experiment.

Dolken 25 hpi total RNA			RNASeq data from this study			Dolken 48 hpi total RNA		
**ORF ID**	**Reads Counts**	**RPKM**	**ORF ID**	**Reads Counts**	**RPKM**	ORF ID	Reads Counts	RPKM
m119.3	5658	73091.9	m168	427096	335276.6	m48.2	2648	99511.4
m119.2	6484	70823.5	m169	251224	269872.5	m48.1	2771	99127.3
m169	5785	58437.5	m119.2	75992	88269.7	m119.3	1903	68076.2
m48.2	4115	55843.7	m119.3	31964	43911.4	m119.2	2217	67058.0
m48.1	4288	55393.8	m119.1	79994	36631.3	m169	2327	65093.1
m168	7013	51769.3	**M116**	153335	33912.4	m168	2889	59056.3
m138	12250	28873.6	m48.1	20856	28651.5	**M94**	2828	30408.4
**M94**	7356	28563.1	m48.2	19175	27672.6	**m106**	1049	26369.6
m119.1	5155	22198.0	**M55**	120762	18433.3	M55	6278	24953.7
**M44**	6300	20543.9	m138	70431	17653.8	**M49**	2664	18456.5
M55	14159	20323.4	m04	27433	14734.7	m119.1	1465	17469.2
M43	8896	19986.3	m15	28880	12618.2	M99	521	17153.4
M99	1480	17596.4	M73	11700	11940.1	M96	599	17142.5
m106	1745	15840.7	M82	44371	10565.7	M85	1385	16515.3
M96	1440	14881.9	m16	15294	10355.9	m120	392	15852.2
M80	7444	14307.7	m06	24835	10166.9	M80	2849	15163.7
M78	4894	13930.3	M83	56249	9921.5	m163	700	14468.2
M49	5144	12869.6	m74	26628	8666.1	m119.5	419	13918.3
m166	3225	11312.8	M99	6687	8454.8	M116	2200	12670.1
m163	1506	11240.7	M78	26868	8132.8	m138	1844	12035.8
M85	2507	10795.4	m14	16884	7987.6	M32	2195	11357.8
m120	703	10266.1	M94	17910	7395.5	M100	1129	11291.2
M116	4727	9830.9	m155	19105	7221.1	M43	1774	11036.8
m119.5	809	9704.4	M49	26770	7122.3	M78	1275	10049.8
M56	5592	9402.9	m160	15359	7101.6	M56	2153	10025.1
m131	818	9393.1	M44	20446	7090.2	M72	1034	9569.4
m25.3	3092	8819.8	M72	18068	6421.5	M44	1043	9418.4
M93	3333	8678.1	M43	26831	6410.4	m166	889	8635.6
m04	1707	8621.7	m106	6024	5815.3	m119.4	213	8430.3
m25.1	3286	8251.9	m125	4405	5721.4	M93	1152	8306.0
M98	3405	8139.9	m152	14736	5555.1	m25.3	999	7891.0
M100	2236	8075.5	M85	12125	5552.3	m25.1	1094	7607.7
m41	820	7925.7	m156	5618	5423.4	M35	1025	7333.5
m148	692	7747.6	M32	26596	5284.9	m25.2	834	7249.6
m25.2	2448	7684.4	m163	6560	5206.9	m25.4	631	7245.6
m25.4	1804	7480.5	m166	13958	5206.8	M121	1289	6860.7
M26	1048	7295.3	m41	4941	5078.7	M73	254	6749.9
m29	1310	7242.8	M114	9027	4903.8	m41	246	6584.3
m147	784	7214.4	m119.5	3805	4853.8	M95	728	6479.6
M114	1411	7207.9	m119.4	3132	4760.4	m74	726	6152.7
M38	2578	6954.9	M84	18376	4465.0	M98	927	6136.7

m160	1587	6900.1	m120	2870	4457.0	M114	423	5983.8
M32	3639	6799.7	M100	11503	4417.9	M53	517	5776.1
M72	2011	6720.9	m148	3552	4229.0	M83	1239	5690.8
m154	1782	6488.2	M25	26622	4076.7	M38	757	5655.3
m29.1	1018	6481.9	m147	4133	4044.5	m131	172	5469.3
m155	1816	6454.5	m131	3152	3849.0	m04	366	5119.1
m119.4	445	6360.2	m03	7494	3823.9	M26	262	5050.5
M92	1062	6176.6	M115	7358	3822.8	m19	197	4952.2
m45.1	5342	6108.1	m25.1	13465	3595.9	m148	158	4898.5
m130	716	6088.3	M80	17278	3531.6	m147	192	4892.6
m145	2166	5963.2	m159	9802	3509.9	m45.1	1527	4834.9
M73	580	5566.0	m05	8263	3462.0	m155	462	4547.2
M53	1297	5232.8	M38	11529	3307.6	M84	691	4372.1
m161	851	5059.0	m08	8023	3210.8	m14	349	4299.4
M95	1563	5023.7	m157	7404	3205.5	m128Ex3	442	4060.3
M35	1800	4650.6	m25.2	9495	3169.6	m29	260	3980.7
m74	1512	4627.3	m145	10727	3140.6	m29.1	215	3790.9
m06	1157	4454.0	M98	12168	3093.4	m130	160	3767.5
M28	1409	4392.1	m25.4	6876	3032.1	M88	422	3676.8
M97	2042	4260.0	M93	10661	2951.9	M103	311	3638.5
M83	2511	4164.9	M37	6828	2819.5	M92	224	3607.7
m128Ex3	1222	4053.7	M56	15652	2798.8	M46	274	3455.6
m19	445	4039.6	M96	2525	2775.0	M28	389	3357.9
M121	2078	3994.0	M50	5753	2592.9	m59	346	3334.9
m13	386	3870.1	m12	4810	2517.3	m13	120	3331.7
M88	1219	3835.4	M103	5540	2489.0	m154	325	3276.8
m156	405	3676.5	m13	2289	2440.6	m156	129	3242.8
M103	867	3663.0	M31	10023	2398.7	m167	380	3235.1
m140	1287	3565.1	m30	12090	2369.5	M37	294	3161.3
M91	358	3562.8	m25.3	7775	2358.5	m161	191	3144.2
M37	908	3525.7	m29	3960	2328.3	M25	774	3086.4
M102	2096	3463.7	M53	5403	2318.1	M69	693	3058.4
m162	405	3400.8	m07	4920	2231.5	M34	685	2980.7
m90	769	3238.7	m154	5463	2115.2	M105	756	2963.8
m17	966	3236.5	m142	6167	2020.9	m159	316	2946.5
m03	659	3162.0	M121	9802	2003.5	m20	637	2883.1
M46	686	3124.2	m17	5614	2000.2	M76	197	2874.2
m142	985	3035.2	m45.1	16325	1985.0	M97	496	2865.4
m20	1813	2963.2	m161	3079	1946.5	M71	230	2852.3
m08	777	2924.1	M35	7079	1945.0	m22	79	2799.2
m139	1364	2841.2	M102	10829	1903.0	m145	366	2790.3
M84	1237	2826.4	M46	3838	1858.8	m90	229	2670.8
m59	805	2801.9	m128Ex3	5156	1818.9	m69.1	86	2666.3
m164	871	2734.1	m20	10461	1818.2	M77	437	2584.8
m14	587	2611.4	M95	5214	1782.1	M91	92	2535.4
m42	304	2490.4	m139	8012	1774.7	m107	157	2517.7
m165	606	2444.9	m123Ex2	444	1714.5	m39	161	2506.2
m137	608	2438.4	M28	5098	1689.9	m21	152	2458.7

m159	708	2384.0	m29.1	2401	1625.8	m160	203	2444.1
m124.1	237	2341.3	m130	1751	1583.4	m126	59	2385.9
M77	1069	2283.3	m19	1571	1516.6	M23	248	2353.7
M79	433	2246.1	m164	4534	1513.5	m165	207	2312.7
M76	421	2218.1	m39	2514	1502.9	m108	116	2307.8
m02	537	2206.3	m42	1699	1480.1	m142	268	2286.9
M31	956	2151.4	M97	6547	1452.5	m15	196	2230.0
m07	500	2132.5	m140	4766	1404.0	m140	290	2224.6
M87	1467	2126.1	M92	2214	1369.4	M75	425	2177.9
m30	1142	2104.6	M88	4030	1348.4	m02	191	2173.1
m152	592	2098.6	m123.1	1132	1347.8	m162	92	2139.2
M71	468	2095.9	m146	3495	1321.0	m40	68	2073.7
m167	673	2069.1	M71	2616	1245.8	M104	392	2068.7
M115	423	2066.6	m124	991	1220.6	m03	155	2059.5
M34	1315	2066.3	M76	2168	1214.7	m124.1	75	2051.7
m22	161	2060.0	m136	2152	1210.5	m164	236	2051.4
m124	167	1934.2	m167	3471	1134.8	m137	178	1976.8
M75	1014	1876.5	m143	3909	1026.6	M115	145	1961.7
M50	426	1805.5	M69	6047	1024.9	M52	265	1903.3
m123.1	160	1791.3	M75	5116	1006.8	m08	182	1896.7
m21	306	1787.4	M26	1343	994.2	M102	400	1830.5
M25	1207	1738.1	m126	639	992.3	M50	155	1819.1
M23	507	1737.6	m158	2438	975.7	M82	289	1792.0
M52	667	1730.0	m127	913	973.5	m139	305	1759.3
m12	345	1697.8	m90	2151	963.4	m06	163	1737.6
m15	403	1655.8	M105	6379	960.4	m123.1	55	1705.2
M69	1030	1641.5	m124.1	907	952.8	m124	52	1667.8
M105	1148	1625.2	m153	2618	921.3	M70	422	1627.0
m127	160	1604.2	m10	1874	916.9	M31	261	1626.5
M104	834	1589.3	m40	772	904.1	m127	58	1610.3
M27	806	1585.5	m69.1	730	869.1	m07	134	1582.6
m05	401	1579.9	M34	5194	867.9	M87	390	1565.2
m143	620	1531.2	m149	1372	852.3	M79	106	1522.6
m39	269	1512.1	M52	3051	841.5	m30	298	1520.8
m40	125	1376.5	m129	903	772.5	m18	423	1511.7
m107	210	1216.1	m165	1794	769.7	m157	134	1510.7
m108	164	1178.3	m02	1711	747.6	m01	51	1405.5
m157	288	1172.5	m21	1178	731.8	m17	148	1373.1
m18	902	1164.1	m137	1701	725.5	M51	80	1271.9
M82	509	1139.7	m135	550	720.9	M24	110	1263.1
m144	325	1137.1	m134	695	719.5	M27	231	1258.3
M24	274	1136.2	M77	3007	683.0	m42	53	1202.3
m69.1	99	1108.4	M91	643	680.5	m12	88	1199.3
m141	416	1098.0	M104	2976	603.1	m16	67	1181.4
M51	174	999.0	m117.1	1905	576.6	m152	108	1060.2
m16	153	974.2	m141	2008	563.6	m143	149	1019.0
m126	61	890.8	M70	3788	560.8	m05	92	1003.7
m01	85	845.9	m09	1144	555.9	m144	96	930.1

m23.1	64	767.7	m11	1160	552.4	m123Ex2	9	905.0
m129	94	756.2	m117	2180	550.3	m146	91	895.7
m146	211	749.9	m22	377	513.0	m129	38	846.6
M70	526	732.3	M27	2440	510.4	m58	52	823.2
m158	179	673.6	m107	809	498.2	m158	78	812.9
m136	124	655.9	m144	1321	491.5	m125	24	811.7
m153	188	622.1	m162	533	475.9	m23.1	24	797.2
m11	118	528.4	m18	3442	472.4	m141	107	782.1
m10	111	510.7	m108	613	468.3	m170	49	772.5
m123Ex2	14	508.4	M87	2873	442.8	m136	43	629.8
m134	51	496.5	M24	925	407.9	m153	67	614.0
m170	83	472.5	M23	1116	406.7	m10	37	471.4
m135	36	443.7	M51	658	401.8	m11	36	446.4
m58	74	423.1	M79	718	396.1	m134	15	404.4
m125	33	403.1	m59	999	369.8	m135	10	341.3
m09	70	319.9	m58	569	345.9	m149	14	226.5
m117.1	106	301.7	m23.1	261	332.9	m09	17	215.1
m149	40	233.7	m151	665	243.6	m117	30	197.2
m117	97	230.2	m150	566	207.9	m117.1	25	197.1
m151	42	144.7	m170	243	147.1	m151	15	143.1
m150	23	79.4	m01	130	137.6	m150	11	105.2

* 8 out of 10 top genes are identical in both lists, bold are divergent
