# Supplementary material for: Dual Analysis of the Murine Cytomegalovirus and Host Cell Transcriptomes Reveal New Aspects of the Virus-Host Cell Interface
Source: PLoS Pathog. 2013 Sep 26;9(9):e1003611. doi: 10.1371/journal.ppat.1003611 (PMC3784481; doi:10.1371/journal.ppat.1003611)
Supplement: Dataset S2 — Spliced cDNA clone overlapping M116 and comparison of predicted protein to current annotation. (PDF) [file ppat.1003611.s002.pdf]

Supplemental File 2. Nucleotide sequence of cDNA clone L29 (2A), Translation of sequence obtained for L29 (2B), and Blast analysis of translated sequence for L29 against predicted MCMV M116 protein sequence (accession # YP_214111) (2C).

Supplemental File 2A. Nucleotide sequence of cDNA clone L29 (sequence from 5' end—Vector sequence is bold; splice junction in red and underlined)

>clone L29

NNNNNNNNNNNNNTTAGCTTGGTACCGAGCTCGGATCCACTAGTCCAGTGTGGTGGAATTCCCGCGGGTTTAAAC~~GAGAACGGCAACGAG
ACGACGGTCGCCGCGCCGGCTCCGCCCGCGGCACCAGCGCCAGCAGCCGCTTCTGCCTCCGCAGCTAGCACTCGGGCCGAGACGCCCGCTGTCGC
CGCCGCCGCCGCTGCCACCACCGCCGCTAAAAAACGGGTTTCGACCATACTATCTCGAGCGAGCGACGGGACGAAGATCTTAACCCCCACCGACG
GGACGAAGATCTTAATCTACGCCTCTTCGTCATCGGACTCTTCGGATATCTCCTCAGACGACTCGGATAGCGAAACGAACTCGTCGTCAAGCGAACC
GTCTAACTCGTCCTTACCACCGGGATCGGAGACCGAACCCTCTTCATCATCCTCATATTCATCTTCATCATCTTCATCATCCTCTCCATCCTCCTCCGAC
TCAGAGGATGAACCATCATCATCATCATCATCGTCATCTACCAGCTCGGACGGCGCTTCATCGGATTCGGAGGCGCCATCTTCGGATTCTGGATCCG
AATCCATAGGCTCGACGTCGCCCAAATCGGAATCGGAGTCCTCAGACTCAGAAAAAGATCAAGAGAAGAATAAGAAAACATCGACACCCGCATCT
ACTCGCGCGAGTTCGCCGCAACCAGGAGCAGCACCAGCAGCCCCCGCGAACACAGAACCGGCCCGAGACGCGTCGACGGAGCTGTCGTCGAAGA
TGTTATCCGCCTCGATCGTGGACAAGCCGACGAAGCTCGCCGACGCTGCCAAGACTACTCCTCAGCAGG**T~~C**AACACAGAGTGCGACAACGGTCC
AGACGGAAAGCGGTCACGCCGGTCTACAGAAAGAGACGCCGAAGAAATCGACGTCCCTATCGACCTTGAAGAGAGCGGGTCGACAGACCAGAGA
ACTGCAGCAGAGCTCGGAGACGGCGACGATCACTGACGCGTCGCGGGTTCCGAGCACGGTCACGGCNGTCCCGCCGACGAGCATCCCGTACCTG
ACNNCACTAACGACAGCCCCGCCAGCACCCACATCAGACGTCGACAACAACACCAACACANCATCACCGCCATCACCACCAAGGACCCGCTACCAC
AGCNGTANNTTCGANAGNATNTNNGANCGTNACTCNCTCNNNNCAGCAGNANTAGNACCNNATCTCNCCTCCNTNCCGTCGAANNCCAGACNN
NACTGNNGNCNCACAGAANGCTACTAATAANNNNANGANNNNNAAAAAANANANNNNNNGCNNATNNCCNCNACNNNNNNNTANNTNCN
NCNTCTGGNNNNNNCCNNNCCNNNNNNNNNGNNNNNNNGGNNNNNTNCNNNNNNNTNCCTNANANNNNNAANNNNNTNCNNNGNNNN
NNGNNGAN

Supplemental File 2B. Translation of sequence obtained for L29. The italicized sequence diverges from published sequence because of change in open reading frame and introduction of stop codon caused by splicing (altered residues due to reading frame shift in red). The 5' end of the transcript has not been unambiguously determined. While current genome annotation indicates M116 is predicted to be 645 AA, These findings provide evidence of a novel truncated protein product that diverges from the predicted protein sequence at residue 350 and introduction of a new stop codon at position 401.

>clone L29: 16 to 984: Frame 1 323 aa
SLVPSSDPLVQCGGIPAGLNENGNETTVAAPAPPAAPAPAAASASAASTRAETPAVAAAAAATTAAKKRVSTIL
SRASDGTKILTPTDGTKILIYASSSSDSSDISSDDSDSETNSSSSEPSNSSLPPGSETEPSSSSSYSSSSSSSSS
PSSSDSEDEPSSSSSSSSTSSDGASSDSEAPSSDSGSESIGSTSPKSESESSDSEKDQEKNKKTSTPASTRAS
SPQPGAAPAAPANTEPARDASTELSSKMLSASIVDKPTKLADAAKTTPQQV**NTECDNGPDGKRSRRSTERDA**
EEIDVPIDLEESGSTDQRTAAELGDGDDHSTOP

Supplemental File 2C. Blast of our translated sequence for L29 against MCMV M116 protein sequence (accession # YP_214111). Note that the spliced clones in the cDNA library are also truncated at the 5' end relative to current gene annotation. While current genome annotation indicates M116 is predicted to be 645 AA, These findings provide evidence of a novel product predicted to be approx. 401 AA.

```
 GENE ID: 3293866 MuHV1_gp103 | UL116 [Murid herpesvirus 1]
(10 or fewer PubMed links)

 Score =  160 bits (405),  Expect = 3e-48, Method: Compositional matrix adjust.
 Identities = 252/255 (99%), Positives = 253/255 (99%), Gaps = 2/255 (1%)
```

```
Query  21   ENGNETTVAAPAPPAAPAPAAASASAASTRAETPAVAAAAAATTAAKKRVSTILSRASDG  80
            +NGNETTVAAPAPPAAPAPAAASASAASTRAETPAVAAAAAATTAAKKRVSTILSRASDG
Sbjct  98   KNGNETTVAAPAPPAAPAPAAASASAASTRAETPAVAAAAAATTAAKKRVSTILSRASDG  157

Query  81   TKILTPTDGTKILIYASSSSDSSDISSDDSDSETNSSSSEPSNSSLPPGSETEPSSSSSY  140
            TKILTPTDGTKILIYASSSSDSSDISSDDSDSETNSSSSEPSNSSLPPGSETEPSSSSSY
Sbjct  158  TKILTPTDGTKILIYASSSSDSSDISSDDSDSETNSSSSEPSNSSLPPGSETEPSSSSSY  217

Query  141  SSSSSSSSSPSSSDSEDEPSSSSSSSSTSSDGASSDSEAPSSDSGSESIGSTSPKSESES  200
            SSSSSSSSSPSSSDSEDEPSSSSSSSSTSSDGASSDSEAPSSDSGSESIGSTSPKSESES
Sbjct  218  SSSSSSSSSPSSSDSEDEPSSSSSSSSTSSDGASSDSEAPSSDSGSESIGSTSPKSESES  277

Query  201  SDSEKDQEKNKKTSTPASTRASSPQPGAAPAAPANTEPARDASTELSSKMETLSASIVDK  260
            SDSEKDQEKNKKTSTPASTRASSPQPGAAPAAPANTEPARDASTELSSKM  LSASIVDK
Sbjct  278  SDSEKDQEKNKKTSTPASTRASSPQPGAAPAAPANTEPARDASTELSSKM--LSASIVDK  335

Query  261  PTKLADAAKTTPQQV  275
            PTKLADAAKTTPQQV
Sbjct  336  PTKLADAAKTTPQQV  350
```
