# Supplementary figures and images for: Dual Analysis of the Murine Cytomegalovirus and Host Cell Transcriptomes Reveal New Aspects of the Virus-Host Cell Interface
Source: PLoS Pathog. 2013 Sep 26;9(9):e1003611. doi: 10.1371/journal.ppat.1003611 (PMC3784481; doi:10.1371/journal.ppat.1003611)

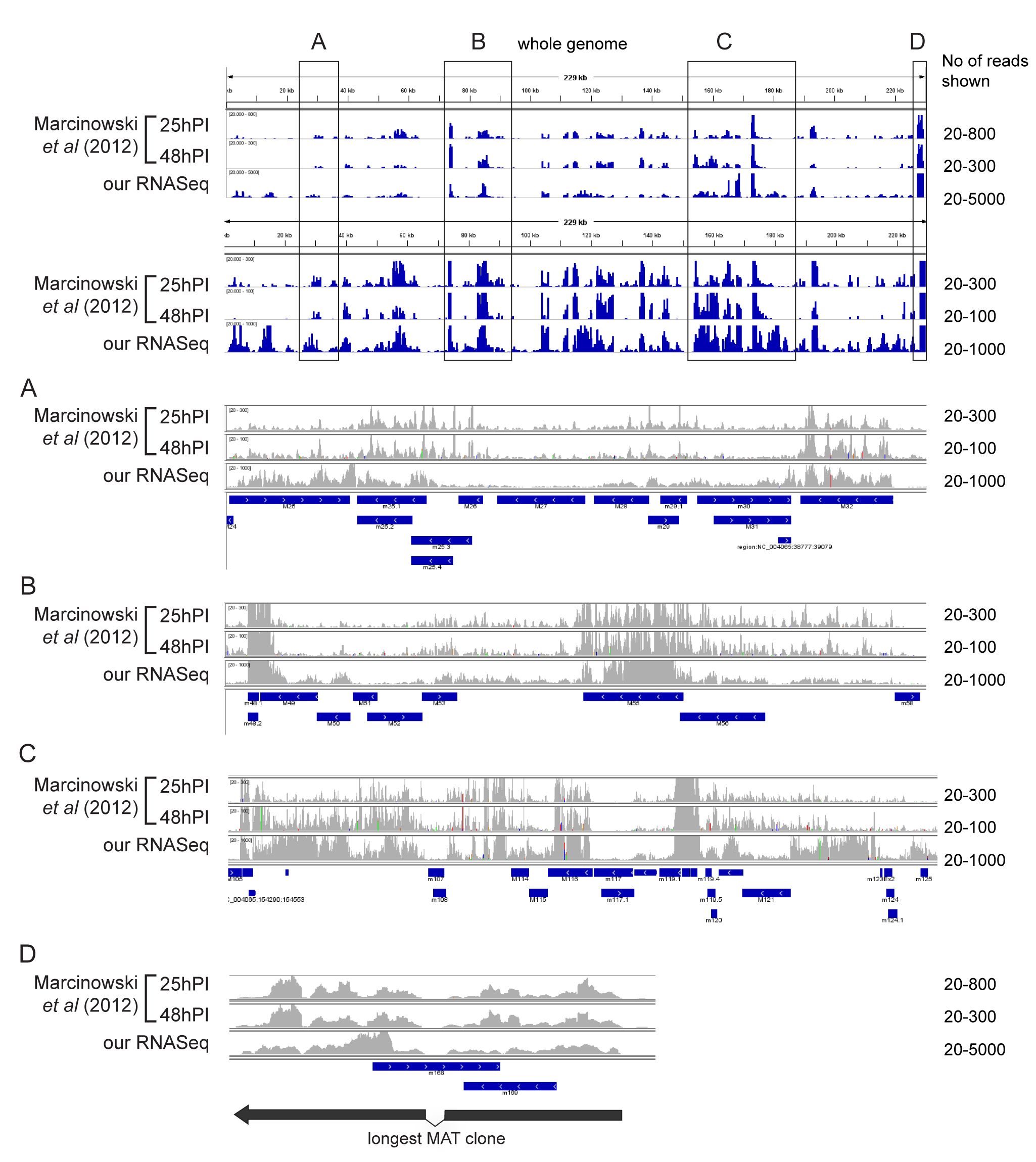

Supplement: Figure S1 — RNA-Seq profiles comparison. RNA-Seq data from total RNA obtained from MCMV infected NIH-3T3 fibroblasts from 25 and 48 hrs PI sequenced by Dölken group (GSE35833) was aligned against MCMV genome (gB acc no NC_004065.1) using Bowtie aligner and visualized in IGV in comparison to our RNA-Seq data. The view of the complete genome is shown at the top with 4 areas magnified below (labeled A–D) and the number of reads displayed are noted on the side. Since viral genes display a wide range of expression levels, the whole genome view is shown in wide data range (upper panel) more suitable for displaying highly transcribed regions and a narrowed data range (lower panel) that is more suitable for less transcribed regions. As can be seen, the profiles of the compared alignments are remarkably similar, the only differences being abundance of certain transcripts which are due to different time points analyzed in comparison to the pooled data of our RNA-Seq and significantly greater depth of at least one order of magnitude of our data in comparison to Marcinowski data. (TIF) [file ppat.1003611.s003.tif]

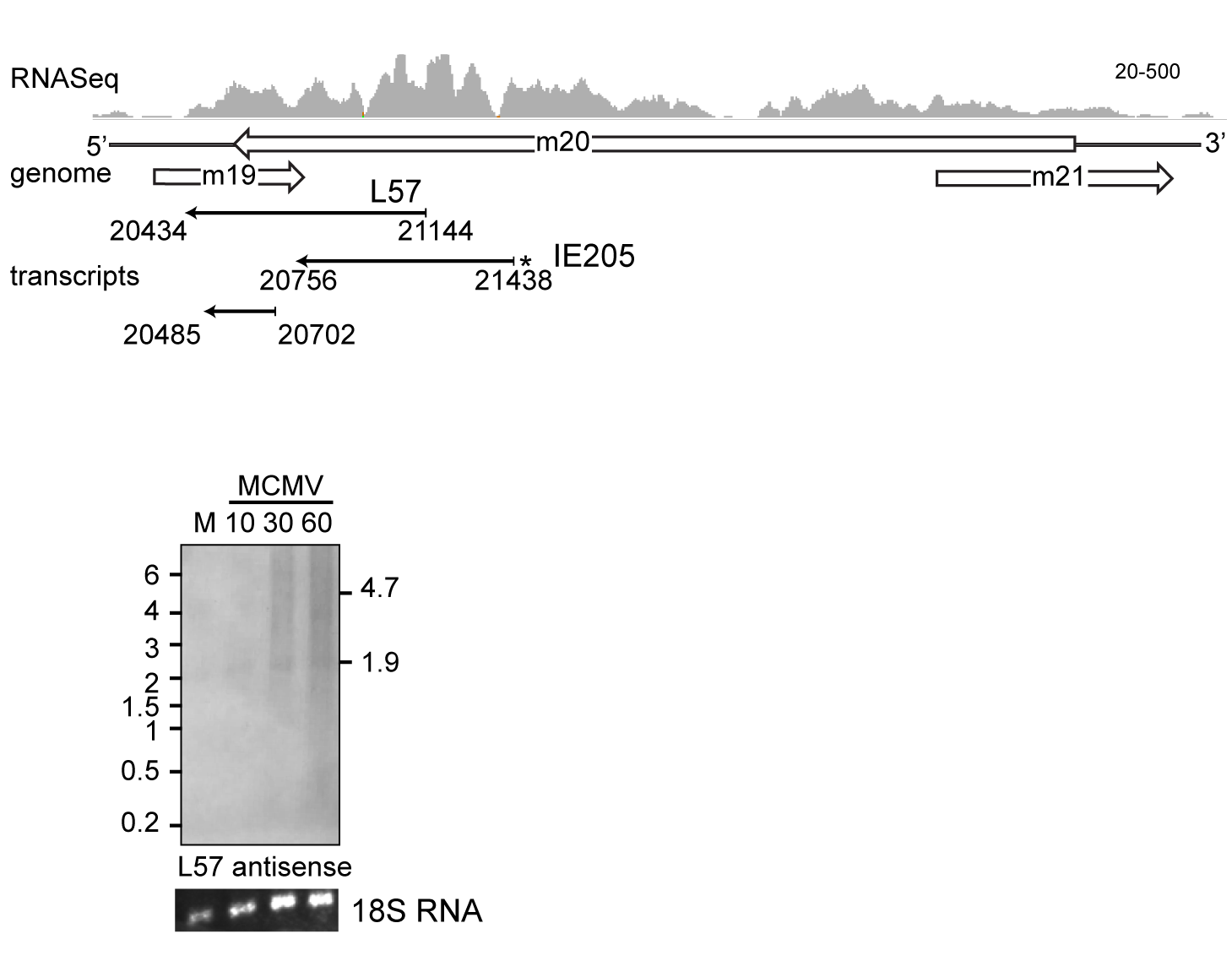

Supplement: Figure S2 — Analysis of the m20–19 region. Balb/c MEF cells were infected with BAC derived Smith virus and harvested 10, 30 and 60 hrs post infection. Total RNA was separated by denaturing gel electrophoresis, transferred to nylon membrane and incubated with probe generated by in vitro transcription from T7 promoter of L57 [A; probe should detect predicted m19(S) transcripts] or probe generated by in vitro transcription from T3 promoter of IE205 transcript [probe should detect m20(S)-m19(AS) transcripts]. RNA integrity and loading was evaluated by inspecting 28S (not shown) and 18S rRNA bands under UV light after transfer to membrane. Predicted genes (Rawlinson's annotation) are depicted as empty arrows, while thin black arrows show longest transcripts cloned in our cDNA library as well as clones used to generate probes (marked with *). 3′ ends of transcripts are marked with arrowheads. The nucleotide coordinates relative to Smith sequence (NC_004065.1) of isolated transcripts are given below thin arrows, while the names of the clones are written above. Gray histograms showRNA-Seqreads aligned to MCMV genome. Maximal possible exposure times were used to ensure even low abundance transcripts are detected and are noted on the blots. (TIF) [file ppat.1003611.s004.tif]

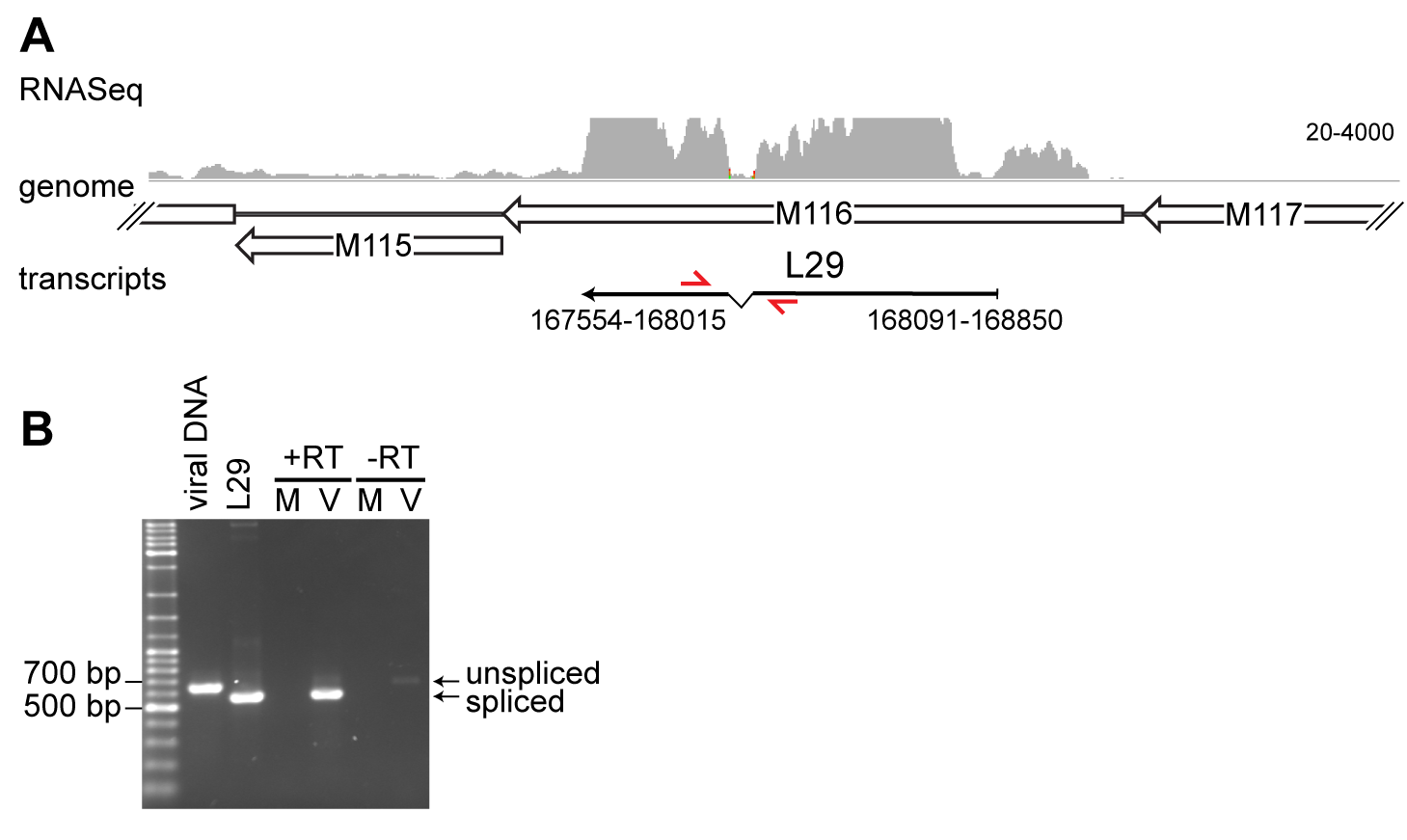

Supplement: Figure S3 — Verification of M116 splicing by PCR. Schematic of the M116 gene region and clone L29 (A) and PCR analysis of the splice site (B). In (A) Predicted genes (Rawlinson's annotation) are depicted as empty arrows, while the thin black arrow depicts clone L29 with red arrows depicting the primers used in (B). The 3′ ends of transcripts are marked with arrowheads. The nucleotide coordinates relative to Smith sequence (NC_004065.1). Gray histograms showRNA-Seq reads aligned to MCMV genome. RNA isolated from WT infected Balb/c MEF used in northern blots was reverse transcribed with oligo-dT primers, and then amplified with primers specific for M116 that flank the putative intron (marked by red arrows). No RT controls were run in parallel. Spliced cDNA clones and viral DNA were used as spliced and unspliced amplification controls, respectively. (TIF) [file ppat.1003611.s005.tif]

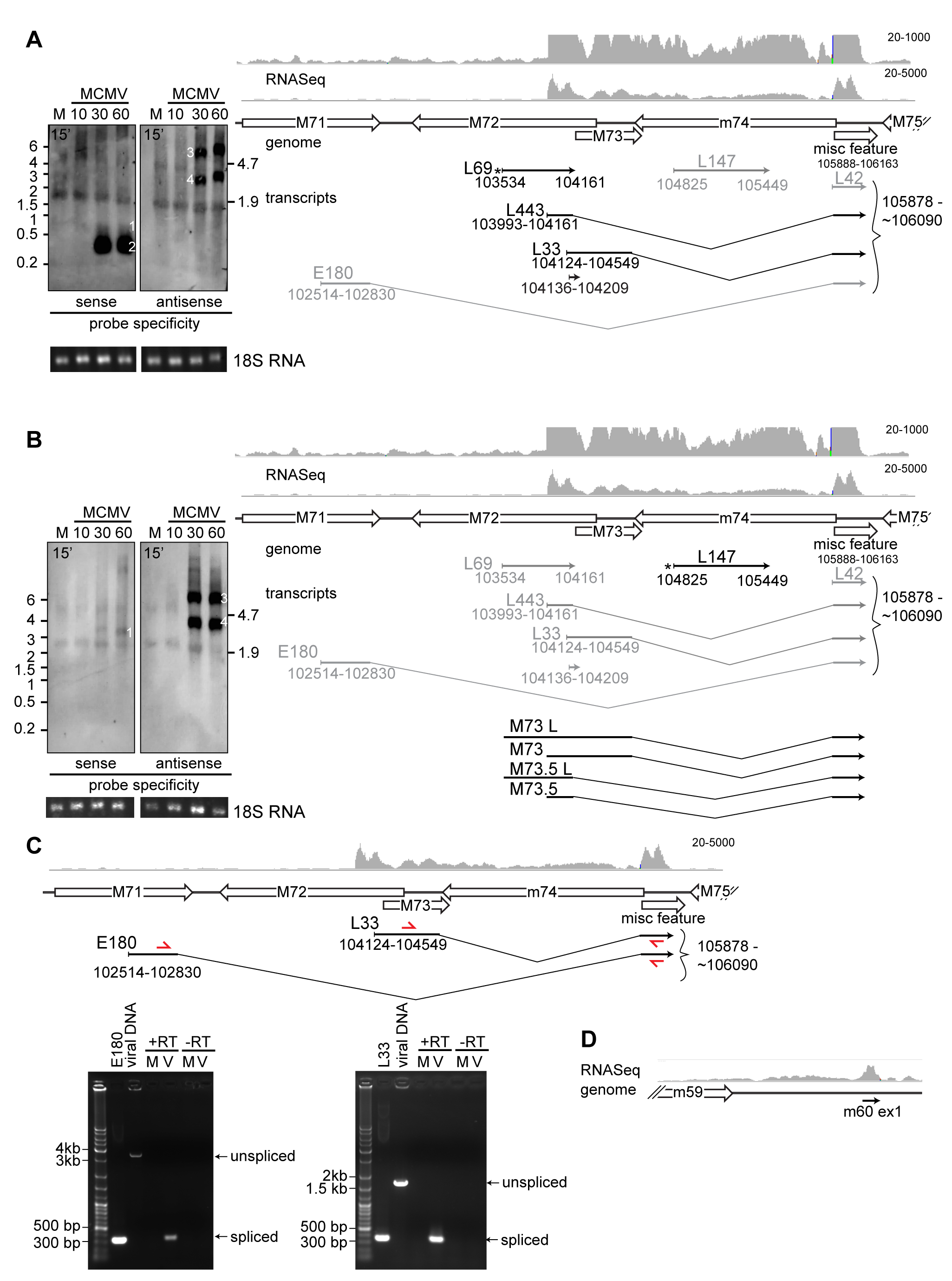

Supplement: Figure S4 — Analysis of the m71-m75 region spliced transcripts by northern blot and PCR. Balb/c MEF cells were infected with BAC derived Smith virus and harvested at indicated times post infection (A and B). Total RNA was separated on denaturing gel electrophoresis, transferred to nylon membrane and incubated with probes specific for S and AS transcripts. RNA integrity and loading was evaluated by inspecting 28S (not shown) and 18S rRNA bands under UV light after transfer to membrane. Predicted genes (Rawlinson's annotation) are depicted as empty arrows, while thin black arrows show longest transcripts cloned in our cDNA library as well as clones used to generate probes (marked with *). Images of northern blots are shown using probes derived from the M72 region in (A) and the m74 in (B). The 3′ ends of transcripts are marked with arrowheads. The nucleotide coordinates relative to Smith sequence (NC_004065.1) of isolated transcripts are given below thin arrows, while the names of the clones are written above. Thin gray lines show isolated transcripts that cannot be detected with the probe. Gray histograms showRNA-Seqreads aligned to MCMV genome. Maximal possible exposure times were used to ensure even low abundance transcripts are detected and are noted on the blots (A and B). In (C), the m71–75 spliced transcript and one of two possible m72–75 spliced transcripts were verified by PCR amplification using primers that flank their putative introns. RNA isolated from WT infected Balb/c MEF used in northern blots was reverse transcribed with oligo-dT primers, and then amplified with primers specific to m71–m75 or m72–m75 spliced transcripts (marked by red arrows). No RT controls were run in parallel. Spliced cDNA clones and viral DNA were used as spliced and unspliced amplification controls, respectively. In (D), the position of exon 1 of m60 Reported by Scalzo et. al. [23], is compared to RNA-Seq data for this genomic region. (TIF) [file ppat.1003611.s006.tif]

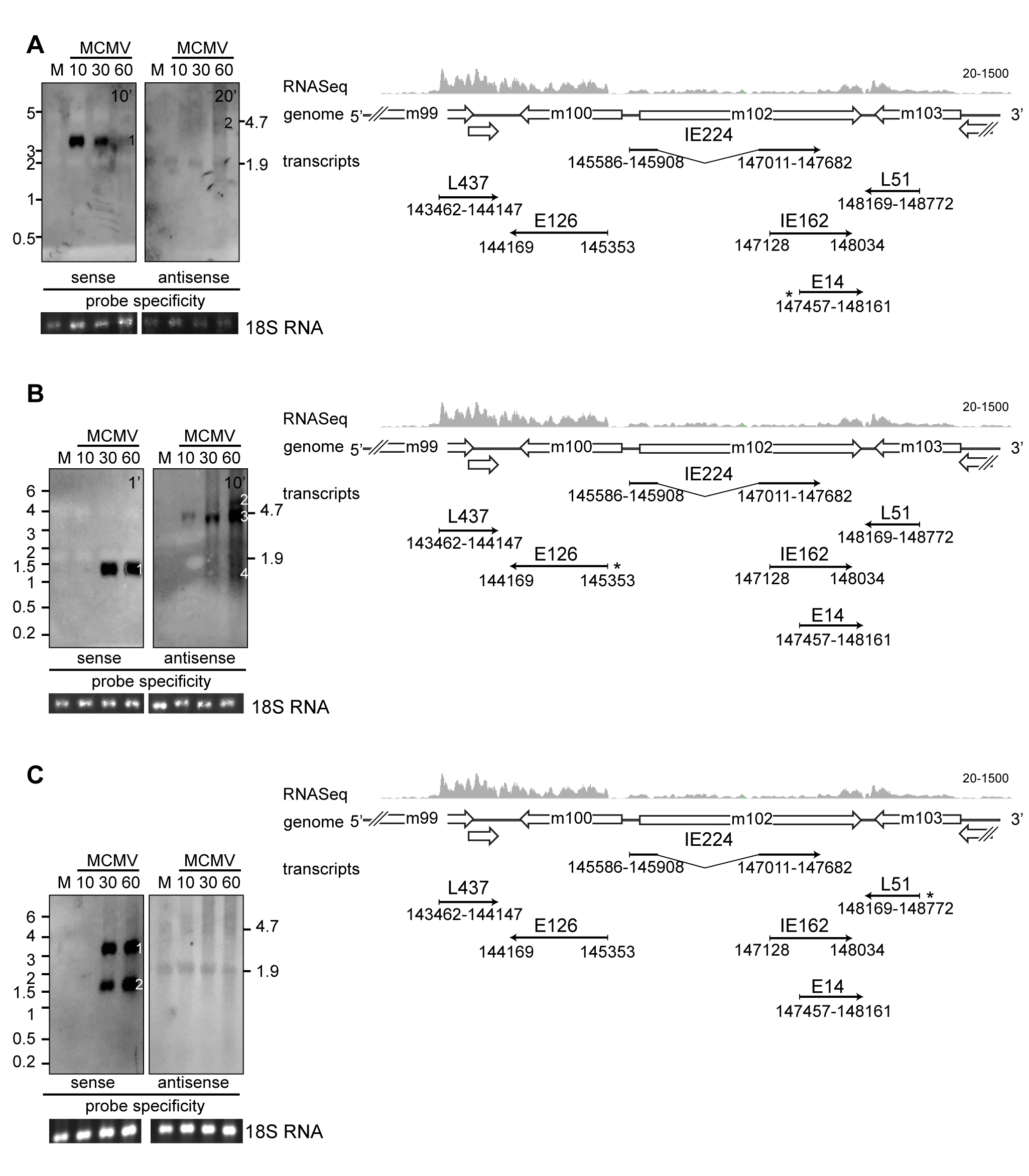

Supplement: Figure S5 — Northern blot analysis of the M100-M103 region. Balb/c MEF cells were infected with BAC derived Smith virus and harvested at indicated times post infection. Total RNA was separated on denaturing gel electrophoresis, transferred to nylon membrane and incubated with probes specific for S and AS transcripts overlapping M102 (A), M100 (B) and M103 (C) genes. RNA integrity and loading was evaluated by inspecting 28S (not shown) and 18S rRNA bands under UV light after transfer to membrane. Predicted genes (Rawlinson's annotation) are depicted as empty arrows, while thin black arrows show longest transcripts cloned in our cDNA library as well as clones used to generate probes (marked with *). 3′ ends of transcripts are marked with arrowheads. The nucleotide coordinates relative to Smith sequence (NC_004065.1) of isolated transcripts are given below thin arrows, while the names of the clones are written above. Thin gray lines show isolated transcripts that cannot be detected with the probe. Gray histograms showRNA-Seqreads aligned to MCMV genome. Maximal possible exposure times were used to ensure even low abundance transcripts are detected and are noted on the blots. (TIF) [file ppat.1003611.s007.tif]

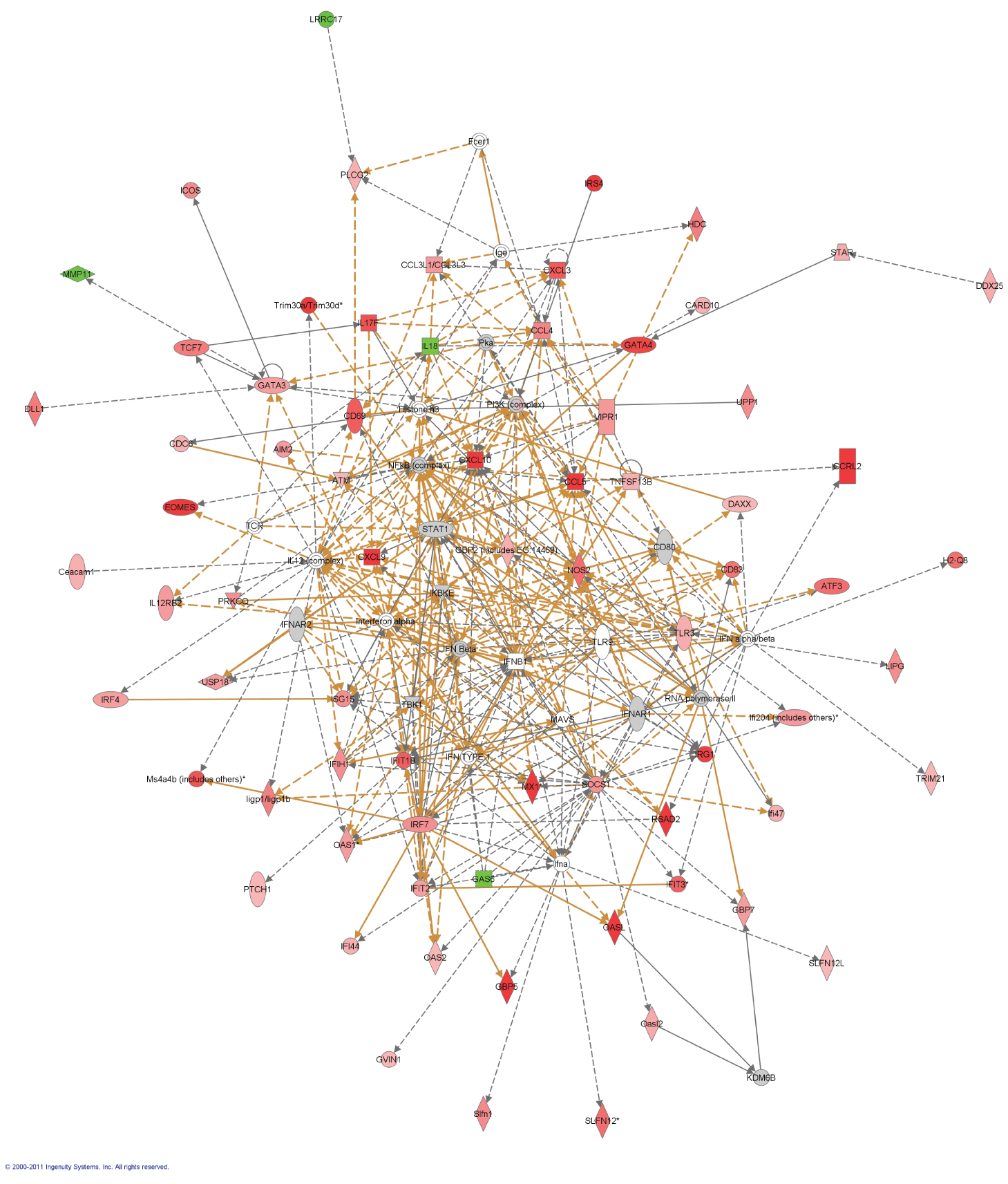

Supplement: Figure S6 — Graphical representation of top 3 genetic networks for differentially regulated genes. Upregulated genes are shown in red, while downregulated are shown in green. Level of differential expression is represented by color saturation with most dramatically changed genes being shown in the most saturated color (strong red or green). These overlapping genetic networks are associated with (i) cell-mediated immune response, cellular development, cellular function and maintenance (30 focus molecules); (ii) infectious disease, antimicrobial response, inflammatory response (27 focus molecules) and (iii) antimicrobial response, inflammatory response, gene expression (24 focus molecules) (see Supplemental table S5). (TIF) [file ppat.1003611.s008.tif]

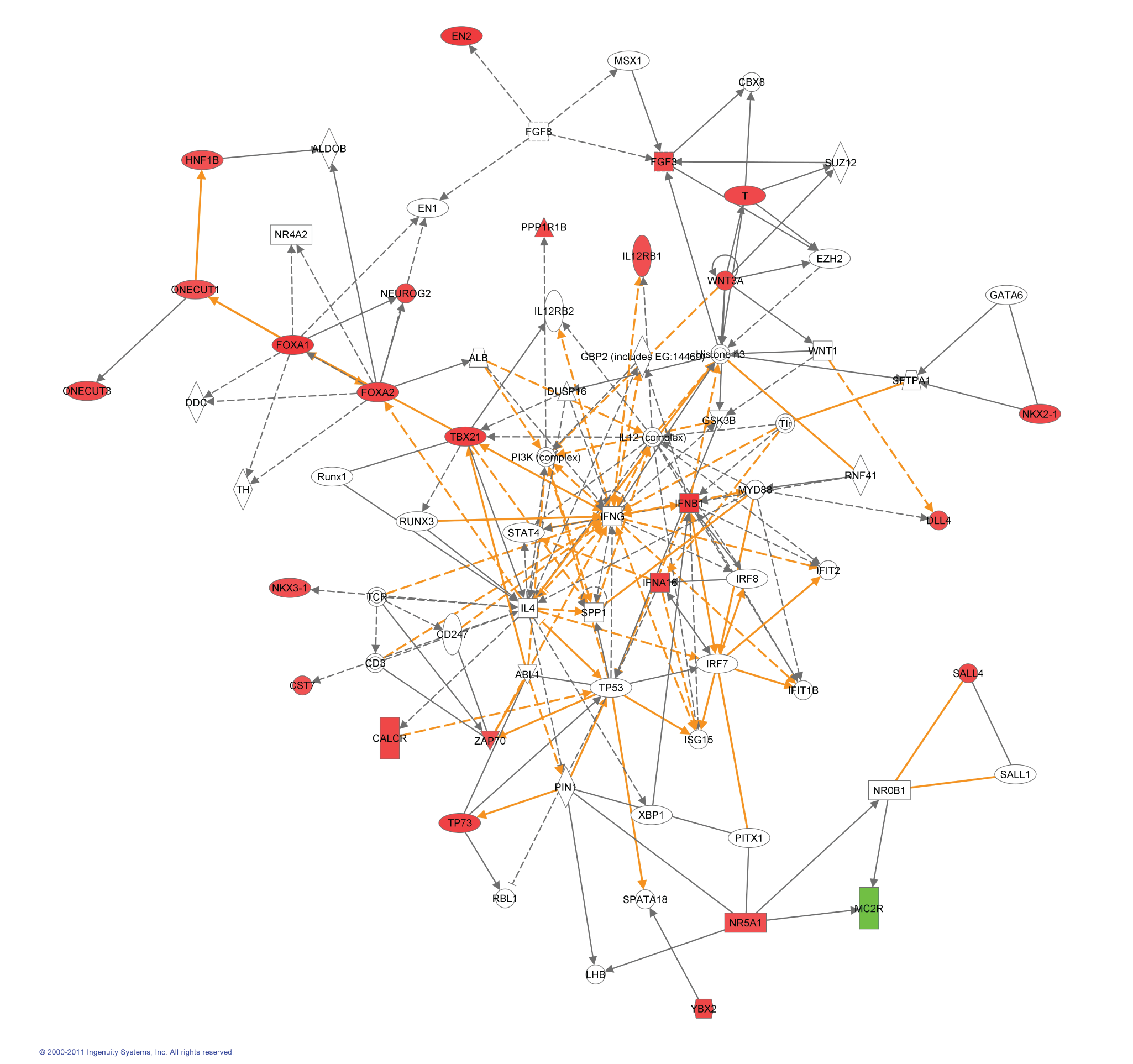

Supplement: Figure S7 — Graphical representation of top 5 genetic networks for genes induced or repressed by infection. Induced genes are shown in red, while repressed are shown in green. Level of differential expression is represented by color saturation with most dramatically changed genes being shown in the most saturated color (strong red or green). These overlapping genetic networks are associated with various developmental processes (see Supplemental table S6). (TIF) [file ppat.1003611.s009.tif]
