# Supplementary material for: Dual Analysis of the Murine Cytomegalovirus and Host Cell Transcriptomes Reveal New Aspects of the Virus-Host Cell Interface
Source: PLoS Pathog. 2013 Sep 26;9(9):e1003611. doi: 10.1371/journal.ppat.1003611 (PMC3784481; doi:10.1371/journal.ppat.1003611)
Supplement: Table S1 — MCMV transcripts identified in this study compared to current NCBI Reference Sequence Gene Annotation. (PDF) [file ppat.1003611.s010.pdf]

Table S1. MCMV Transcripts Identified in This Study Compared to Current NCBI Reference Sequence Gene Annotation

Gene ID NC_004065.1 (MuHV1)	Overlapping Genes (orientation[1])	Strand	No. of Clones	Start[2]	End[2]	Range start[3]	Range end[3]	ORF Length[4]	Libraries
gp004	m04 (S)	+	7	3250	4102	3250	4102	852	4IE,E,L
gp006	m06 (S)	+	6	5319	6260	5319	6365	941	4IE,E,L
gp008	m08 (S)	+	1	7679	8440	7679	8440	761	L
gp015, gp016	m15, m16 (S) spliced	+	1	14635, 15622	15083, 15700	14635, 15622	15083, 15700	448+78	E
	m15, m16 (S)	+	4	14027	15700	14772	15699	1673	2E, l, IE
gp017	m17 (S)	-	2	16032	15704	16032	15704	328	IE
gp018	m18 (AS) spliced	+	1	17079, 17853, 18351	17188, 17957, 18777	17079, 17853, 18351	17188, 17957, 18777	109+104+426	L
gp018	m18 (AS)	+	1	18927	19285	18927	19285	358	L
gp019	m19 (AS)	-	1	20702	20485	20702	20485	217	L
gp020, gp019	m20 (S), m19(AS)	-	3	21144	20434	21438	20434	710	IE,E,L
gp026	M25(S)	+	6	27240	28285	26206	28959	1045	4L,2E
gp027, gp028	m25.1 (S)	-	3	29893	29169	30293	29169	724	2E,1L
gp027, gp028	m25.1, m25.2 (S)	-	1	30321	29128	30321	29128	1193	E
gp34	m29 (S), m29.1 (AS)	-	1	36135	35849	36135	35849	286	E
gp036	m30, M31 (S)	+	1	37055	37626	37055	37626	571	E
gp038	M32(S)	-	6	40210	39324	40886	39195	886	3E, 3L
gp039	M34 (S) spliced	+	1	44012, 44304	44242, 44516	44012, 44304	44242, 44516	230+212	E
gp040	M35 (S)	+	2	47052	47522	47052	47522	470	E
gp040, IGR[5]gp040-gp041	M36, M36 Ex2 (S/AS)	-	1	47794	47533	47794	47533	261	L
gp041	M37 (S)	-	2	50148	49411	50148	49391	737	1IE, 1L
gp045	m41 (S)	-	2	54217	53677	54217	53677	540	L
gp045, gp046	m42, m41 (S)	-	4	54863	53699	54863	53678	1164	3L, IE
gp045, gp046	m42, m41 (S) spliced	-	1	55312, 54218	55123, 53678	55312, 54218	55123, 53678	189+540	IE

IGR[5]gp046-047, gp047	m42 (S)	-	1	54842	54508	54842	54508	334	1L
gp047	M43 (S)	-	9	56402	55336	57157	55336	1066	6E, 2L, 1IE
gp047, gp048	M44, M43 (S) spliced	-	1	58976, 57157, 56667	58668, 56856, 56361	58976, 57157, 56667	58668, 56856, 56361	308+301+306	IE
IGR gp048-gp049	M45, M44 (S)	-	1	59414	59271	59414	59271	143	L
IGR gp048-gp049, gp049	M45 (S)	-	1	60126	59270	60126	59270	856	E
gp051	M47(AS), M46(S)	-	1	63968	63506	63968	63506	462	L
gp053	m48.2 (S), m48.1 (AS)	-	3	73939	73535	73939	73526	404	2L,1E
gp053, gp054	M49 (S), m48.2 (S), m48.1 (AS)	-	18	74312	73526	74312	73526	786	10L, 8E
gp054	M49 (S)	-	2	74304	73885	74818	73885	419	1E, 1L
gp054	M50, M49 (S)	-	1	75506	74851	75506	74851	655	L
gp058	M53 (S)	+	4	78853	79534	78853	79534	681	3L, 1E
gp058 AS	M53 (AS)	-	1	80332	79485	80332	79485	847	IE
IGR gp058-gp059	M54 (S)	-	2	80561	79589	80561	79589	972	1IE, 1L
go059, IGR gp058-gp059	M55, M54 (S)	-	2	84005	82893	84005	82893	1112	L
gp060	M69 (S)	-	1	96916	96080	96916	96080	836	E
gp066, IGR AS gp069-gp070	M71, IGR m74-M75 (S)	+	1	102514, 105879	102830, 106090	102514, 105879	102830, 106090	316+211	E
gp067	M72(AS), M73(S)	+	2	103709	104265	103534	104265	556	1E, 1L
gp067, IGR gp069-gp070	M72 (S), M73(AS), IGR m74-M75	+	4	103993, 105878	104161, 106090	103993, 105878	104161, 106095	168+213	L

gp068, IGR gp069-gp070	M72 (S), M73(AS), IGR m74-M75 (alternate splice)	+	1	104124, 105878	104549, 106089	104124, 105878	104549, 106089	425+211	L
gp068	M72 (AS)	+	1	104136	104209	104136	104209	73	L
gp069	M74 (AS)	+	1	104825	105449	104825	105449	624	L
IGR gp069-gp070	IGR m74-M75	+	1	105878	106095	105878	106095	217	L
gp071,gp073	M76(AS), M78(S)	+	1	108476, 111789	108714, 112145	108476, 111789	108714, 112145	238+356	E
gp073	M78 (S)	+	8	111752	112593	110933	111866	841	7IE, 1L
gp073	M78 spliced (S)	+	1	111280, 111444	111409, 111710	111280, 111444	111409, 111710	129+266	E
IGR gp073-gp074	M79 (AS)	+	1	112418	112595	112418	112595	177	L
gp075	M80 (S)	+	8	114322	115140	113589	115524	818	4E, 2L, 2IE
gp075	M80 spliced (S)	+	1	114889, 115187	115148, 115396	114889, 115187	115148, 115396	259+209	L
gp076	M82 (S)	-	4	117413	116486	117413	115526	927	3E, 1L
IGR gp078-gp079, gp079	IGR M84-85, M85 (S)	-	2	122735	121931	122735	121931	804	L
gp081	M88 (S)	+	1	131047	131370	131047	131370	323	L
gp084, gp085	M92 , M93, M94 (S)	+	1	134691, 135956	135369, 136399	134691, 135956	135369, 136399	678+443	L
gp085	M93 (S)	+	1	135978	136146	135978	136146	168	L
gp085, gp086 spliced 1	M93, M94 spliced1 (S)	+	1	135978, 136181	136052, 136754	135978, 136181	136052, 136754	74+573	L
gp085, gp086 spliced 2	M93, M94 spliced2 (S)	+	1	135978, 136651	136524, 137227	135978, 136651	136524, 137227	546+576	L
gp085, gp086	M93, M94 (S)	+	4	136264	137333	135978	137333	1069	2L, IE, E
gp086	M94 (S)	+	3	136587	137345	136487	137345	758	2L, E
gp088, gp089	M95, M96 (S)	+	1	139307	139980	139307	139980	673	L
gp089	M96 (S)	+	1	139628	139967	139628	139967	339	E
IGR gp089-gp099, gp099	M97 (S)	+	1	139995	140880	139995	140880	885	IE

gp092	M98, M99 (S)	+	8	143499	144148	143462	144150	649	5L, 3E
gp093	M100 (S)	-	6	145355	144276	145355	144276	1079	3E, 3L
gp094	M102 spliced (S)	+	1	145586, 147011	145908, 147682	145586, 147011	145908, 147682	322+671	IE
gp094	M102 (S)	+	3	147128	148034	147128	148169	906	2E, IE
gp095	M103 (AS)	-	2	148772	148169	148772	148169	603	L
gp097	M105 (AS)	+	2	153268	153874	153268	153874	606	L
gp098	m106 (S)	-	1	154101	154073	154101	154073	28	E
gp098, IGR gp098-gp099	IGR m106-m107, m106 (S)	-	1	161719	153867	161719	153867	7852	L
gp098, IGR gp098-gp099	IGR m106-m107, m106 spliced (S)	-	3	161919, 154368	161622, 153886	161904, 154368	161622, 153886		3E, 1L
IGR gp098-gp099	IGR m106-107	-	1	161357	160933	161357	160933	424	L
IGR gp098-gp099, gp099	m108 (S)-m107 (AS)	-	1	162228	160670	162228	160670	1558	E
gpM112, gpM113	M112, M113 spliced (S)	+	1	163778, 163983	163891, 164157	163778, 163983	163891, 164157		E
gpM112, gpM113	M112 Ex1, M113, M112 Ex2, M112 Ex3 (last exon in IGR M112 Ex3-M114) (S)	+	1	163779, 163983, 164485, 164871	163891, 164160, 164582, 165510	163779, 163983, 164485, 164871	163891, 164160, 164582, 165510	112+177+97+639	L
gpM113	IGR-m112Ex3-M114 (S)	+	1	164516	164581	164516	164581	65	E
gpM113	M113 (S)	+	1	164877	165502	164877	165502	625	L
gpM113, IGR gpM113, gp101	M113, M114 (S)	+	1	165020	165504	165020	165504	484	IE
gp101	M114 (S)	-	1	166087	165497	166087	165497	590	L
gp101, gp102	M114, M115 (S)	-	1	166679	165935	166679	165935	744	L

gp103	M116 spliced (S)	-	8	168850, 168015	168091, 167554	168685, 168015	168091, 167555	759+461	5E, 3L
gp103	M116 (S)	-	15	169140	168095	169140	167261	1045	9L, 6E
gp106	m119, M118 (S)	-	1	171957, 171585	171684, 171255	171957, 171585	171684, 171255	273+330	E
gp107, gp108	m119.1, (S)	-	1	173217	172789	173217	172789	428	L
gp107, gp108, gp109, IGR gp109-gp110	m119.3, m119.2, m119.1(S)	-	7	173897	172792	173897	172792	1105	4E, 2L, 1IE
gp108, gp109 (AS)	m119.2, (S) m119.3 (AS)	+	1	173154	173578	173154	173578	424	IE
gp107, gp108, IGR gp108-gp109	m119.3, m119.2 (S)	-	10	173899	172973	173905	172790	926	6L, 4E
gp109	m119.3 (S)	-	2	173892	173576	173892	173576	316	E
IGR gp108-gp109	IGR m119.3 - m119.4 (S)	-	1	173902	173737	173902	173737	165	E
gp108, gp109, gp110, gp111 (AS), gp112	m120(S), m119.5(AS), m119.4 (S),m119.3(S), m119.2 (S)	-	2	174546	173131			1415	IE, E
gpM122Ex5	M122 Ex5 (S)	-	1	178405	177900	178405	177900	505	L
gpm123Ex4	m123 Ex4 (S)	-	2	180323	179554	180323	179554	769	L
gpM122Ex5, gpm123Ex4, gpm123Ex3, gp114, gp114ex2, gp115	IGR (m124.1 and m125), m123Ex2, m123 Ex3,m122 Ex5 (S)	-	1	182798, 181562, 181770, 179520	182596, 181371, 181659, 179420	182798, 181562, 181770, 179520	182596, 181371, 181659, 179420	202+191+111+100	E
gp121, gp122	m131 (S) - m129 (AS)		1	188054	187318	188054	187318	736	E
gpm132Ex2, gp124	m133 Ex1, m132 Ex2, m131 (S)	-	3	189791, 188602	188880, 188407	189791, 188602	188880, 188407	991+195	2IE, 1L

gp123, gpm132Ex2	m132 Ex2 - m131 (S)	-	2	188885, 188603	188695, 188292	188885, 188603	188695, 188292	190+311	E, L
gp124	m133 Ex1 (S)	-	1	189793	188949	189793	188949	844	IE
gp128	m137 (AS)	+	1	191105	191373	191105	191373	268	IE
IGR gp128-gp129, gp129	m138, m137 (S)	-	1	193025	192162	193025	192162	863	L
gp129	m138 (S)	-	12	193289	192188	193986	192160	1101	6E, 3IE, 3L
gp130	m139 (S)	-	1	194810	194091	194810	194091	719	IE
gp133	m142 (S)	-	2	200648	199635	200648	199635	1013	IE, L
IGR gp135, gp136	m145 (S)	-	6	204650	203973	204650	203973	677	3E, 3L
gp140	m149 (AS), m150 (S)	-	1	208012	207467	208012	207467	545	L
gp141, gp142	m150, m151 (AS)	+	1	208477	210069	208477	210069	1592	IE
gp142	m151 (S)	-	1	209564	208963	209564	208963	601	E
gp145	m154 (S)	-	1	213864	212909	213864	212909	955	IE
gp146	m155 (S)	-	3	215486	214468	215486	214373	1018	2IE, L
gp147	m156, m155 (S)	-	2	215873	215098	215873	215098	775	E
IGR gp149-gp150, gp150	m159 A (S)	-	1	218327	218054	218327	218054	273	L
gp150	m159 B (S)	-	1	219397	219132	219397	219132	265	L
gp151	m160 (S)	-	1	219890	219460	219890	219460	430	L
gp151	m160, m161 (S)	-	1	220641	219677	220641	219677	964	L
gp154	m163 (S)	-	1	222281	221832	222281	221832	449	L
gp154	m164 - m162 (S)	-	1	222465	221878	222465	221878	587	IE
gp154, gp155	m164, m163 (S)	-	14	222616	221986	222616	221832	630	10L, 3E, 1IE
gp157	m166 (S)	-	5	225639	224735	225650	224331	904	3IE, E, L
gp157, gp158	m167, m166 (S)	-	1	226145	225250	226145	225250	895	L

gp158, gp159, gp160	IGR m167-m168, m168 (AS), m169 (S), IGR m169-m170		138	229086, 228247	228325, 227426	229112, 228247	228325, 227426	761+821	28IE, 57E, 53L

[1] Sense (S) or antisense (AS) relative to annotated gene.
[2] Start and end values were derived from the longest clone in the group.
[3] Start range and end range were derived from all clones belonging to a group.
[4] Based on longest clone; Plus signs indicate spliced genes and exon lengths are given.
[5] IGR, Intergenic region
