# Supplementary material for: Dual Analysis of the Murine Cytomegalovirus and Host Cell Transcriptomes Reveal New Aspects of the Virus-Host Cell Interface
Source: PLoS Pathog. 2013 Sep 26;9(9):e1003611. doi: 10.1371/journal.ppat.1003611 (PMC3784481; doi:10.1371/journal.ppat.1003611)
Supplement: Table S4 — Spliced transcripts of MCMV. (PDF) [file ppat.1003611.s013.pdf]

Table S4. Spliced transcripts of MCMV#

Overlapping Gene(s) or Known Designation	Strand	Clone Name	Total No. of Clones Found in This Study	Genomic Position (only positions of longest clone shown)ǂ	First Report
**#m15, m16**	+	E253	1	14635-15083 + 15622-15700	This Study
**m18**	+	L123	1	17079-17188 + 17853-17957 + 18351-19285	This Study
**M33**				41486-41519 + 41679-42780	Poynter et al. [2]
**M34**	+	E196	1	44012-44242 + 44304-44516	This Study
**M36**	-			49267-49036 + 48909-47621	Rawlinson et al.[1]
**m42, m41**	-	IE106	1	55312-55123 + 54218-53678	This Study
**M44, M43**	-	IE160		58976-58668 + 57157-56856 + 56667-56361	This Study
**m60**				94984-95063 + 105879-106093	Scalzo et al.[6]
**#M71, IGR m74-M75**	+	E180	1	102514-102830 +105879-106090	This Study
**#M73**	+	L33	1	103985-104548+ 105879-106093	Scalzo et al.[6] and this study
**M73.5**	+	L443	4	103985-104160+ 105879-106093	Scalzo et al.[6] and this study
**M73.5 long**	+			103700-104160+ 105879-106093	Scalzo et al.[6]
**M76(AS), M78(S)**	+	E139	1	108476-108714 + 111789----112593*	This Study
**M78**	+	E41	1	111280-111409 + 111444----112592*	This Study
**M80**	+	L116	1	114889-115148 + 115187-115396	This Study
**M92 , M93, M94**	+	L14	1	134691-135369 + 135956-137333*	This Study
**M93, M94**	+	L107	1	135978-136052 + 136181-136754	This Study
**M93, M94**	+	L172	1	135978-136524 + 136651-137227	This Study
**M89**	-			138283-137393 + 132771-131649	Rawlinson et al.[1]
**M102**	+	IE224	1	145586-145908 + 147011-147682	This Study
**Stable 7.2kb intron**				162090-161622 + 154365-153916	This Study and Kulesza and Shenk[3].

**Stable 8.0kb intron**				162606-162415 + 154365-153916	Kulesza and Shenk [3]
**Stable 7.2kb intron IGR m106-m107, m106(S)**	-	E289<br>E206<br>L63	3	161905-161622 + 154368-153873, 161919-161622 + 154368-153886, 161904-161622 + 154368----153867*	This Study and Kulesza and Shenk[3]
**M112**	+			163097-163889 + 163983-164159 + 164486-164505	Rawlinson et al.[1] Ciocco-Schmitt et al.[5] demonstrate 4 transcripts (each with 3 exons) of 1.5, 1.4, 1.1 and 0.9kb, but genomic positions were not provided.
**M112 Ex1, M113, M112 Ex2**	+	E184	1	163778-163891 + 163983-164157	This Study
**M112 Ex1, M113, M112 Ex2, M112 Ex3 (last exon in IGR M112 Ex3-M114)**	+	L2	1	163779-163891 + 163983-164160 + 164485-164582 + 164871-165510*	This Study
**#M116**	-	E367, E263, L214, E110, L243, E231, E135, L29	8	168189-168091 + 168015-167555	This Study
**m119, M118**	-	E243		171957-171684 + 171585-171255	This Study and Rawlinson et al.[1]
**M102**		IE224		145586-145908 + 147011-147682	This Study
**m123 Ex2,3,4**				181766-181660 + 181562-181372 + 181249-179763	Rawlinson et al.[1]
**M122 Ex 5**				179517-177983	Rawlinson et al.[1]
**M128 Ex3**				186085-187296	Rawlinson et al.[1]
**m133 Ex1, m132 Ex2**	-			189795-188881 + 188601-188382 +	Lagenaur et al.,[4] Rawlinson et al.[1] (Smith strains has alternate donor site at 188840)
**m133 Ex1, m132 Ex2, m131**	-	IE138	1	189808-189499 + 188602-188269	This Study
**IGR m124.1 - m125, m123Ex2, m123 Ex3, m122 Ex5**	-	E279	1	182798-182596 + 181770-181659 + 181562-181371 + 179520-179420	This Study
**m133 Ex1, m132 Ex2, m131**	-	L78<br>IE208	2	189791-188880 + 188602-188407*	This Study

**m132 Ex2 - m131**	-	E96<br>L102	2	188885-188695 +<br>188603-188292*	This Study
**m165, m164, m163**	-	IE197	1	223828-223662 +<br>223593----221832*	This Study
**[#]m169(S) m168 (AS)**	-	E20,<br>E125	139	229112-228325<br>+228247-227426	This Study

[#] The spliced MAT transcript [m169(S) m168 (AS)], the spliced M116 transcript represented by clone L29, and the spliced transcripts in the M71-M75 region represented by clones L33 and E180 have been verified by other methods (see Results). The spliced transcript in the m15-16 region could not be validated by PCR (see Results). Other novel spliced transcripts isolated in the cDNA library of this study have not yet been validated by other methods.
*Denotes 3' end of clone was successfully sequenced.
ǂ Genomic positions determined by aligning sequences of the clones to target genome using MegaBLAST.

[1]Rawlinson WD, Farrell HE, Barrell BG. Analysis of the complete DNA sequence of murine cytomegalovirus. J Virol. 1996 Dec;70(12):8833-49.

[2] Identification and characterization of a G protein-coupled receptor homolog encoded by murine cytomegalovirus. Davis-Poynter NJ, Lynch DM, Vally H, Shellam GR, Rawlinson WD, Barrell BG, Farrell HE. J Virol. 1997 Feb;71(2):1521-9.

[3] Murine cytomegalovirus encodes a stable intron that facilitates persistent replication in the mouse.Kulesza CA, Shenk T.Proc Natl Acad Sci U S A. 2006 Nov 28;103(48):18302-7

[4]Lagenaur LA, Manning WC, Vieira J, Martens CL, Mocarski ES. Structure and function of the murine cytomegalovirus sgg1 gene: a determinant of viral growth in salivary gland acinar cells. J Virol. 1994 Dec;68(12):7717-27.

[5]Ciocco-Schmitt GM, Karabekian Z, Godfrey EW, Stenberg RM, Campbell AE, Kerry JA. Identification and characterization of novel murine cytomegalovirus M112-113 (e1) gene products. Virology. 2002 Mar 1;294(1):199-208.

[6]Scalzo AA, Dallas PB, Forbes CA, Mikosza AS, Fleming P, Lathbury LJ, Lyons PA, Laferté S, Craggs MM, Loh LC. The murine cytomegalovirus M73.5 gene, a member of a 3' co-terminal alternatively spliced gene family, encodes the gp24 virion glycoprotein. Virology. 2004 Nov 24;329(2):234-50.
